# The CRY1–COP1–HY5 axis mediates blue-light regulation of *Arabidopsis* thermotolerance

**DOI:** 10.1016/j.xplc.2025.101264

**Published:** 2025-01-29

**Authors:** Siyuan Liu, Qiongli Wang, Ming Zhong, Guifang Lin, Meiling Ye, Youren Wang, Jing Zhang, Qin Wang

**Affiliations:** 1 College of Life Sciences, Basic Forestry and Proteomics Research Center, Fujian Agriculture and Forestry University, Fuzhou 350002, China; 2 Key Laboratory of Ministry of Education for Genetics, Breeding and Multiple Utilization of Crops, College of Agriculture, Fujian Agriculture and Forestry University, Fuzhou 350002, China

**Keywords:** blue light, heat stress, thermotolerance, CRY1, HY5, HSF

## Abstract

High-temperature stress, also referred to as heat stress, often has detrimental effects on plant growth and development. Phytochromes have been implicated in the regulation of plant heat-stress responses, but the role of blue-light receptors, such as cryptochromes, in plant blue-light-dependent heat-stress responses remains unclear. We found that cryptochrome 1 (CRY1) negatively regulates heat-stress tolerance (thermotolerance) in *Arabidopsis*. Heat stress represses CRY1 phosphorylation. Unphosphorylated CRY1 exhibits decreased activity in suppressing the interaction of CONSTITUTIVE PHOTOMORPHOGENIC 1 (COP1) with ELONGATED HYPOCOTYL 5 (HY5), leading to excessive degradation of HY5 under heat stress in blue light. This reduction in HY5 protein levels subsequently relieves its repression of the transcription of HY5 target genes, especially the heat-shock transcription factors. Our study thus reveals a novel mechanism by which CRY1-mediated blue-light signaling suppresses plant thermotolerance and highlights the dual function of the CRY1–COP1–HY5 module in both light- and heat-stress signaling, providing insights into how plants integrate heat stress and light signals to optimize their survival under heat stress.

## Teaser

This study reports a CRY1–-COP1–-HY5 module that mediates blue-light regulation of *Arabidopsis thermotolerance.*Heat stress represses the blue-light-dependent phosphorylation of CRY1. Unphosphorylated CRY1 shows decreased activity in suppressing the COP1–HY5 interaction, leading to excessive degradation of HY5 under heat stress in blue light. This, in turn, alleviates HY5 repression of *HSF* transcription.

## Introduction

Light is one of the most important environmental cues that regulate plant growth and development. Light signals are perceived by different photoreceptors. Cryptochromes (CRYs) are photolyase-like flavoproteins that serve as blue-light receptors in plants. Plant CRYs undergo light-induced photooligomerization to become photoactivated in response to blue light (Wang et al., 2016; Liu et al., 2020). Once photoactivated, CRYs further condense into photobodies in the nucleus, which have been identified as liquid–liquid phase separation droplets (Yu et al., 2009; Wang et al., 2021). Photoactivated CRYs undergo blue-light-dependent phosphorylation by four closely related photoregulatory protein kinases (PPK1 to PPK4) (Liu et al., 2017; Gao et al., 2022) and are subsequently polyubiquitinated by two E3 ubiquitin ligases, Cul4^COP1/SPAs^ ligase and Cul3^LRBs^, leading to their degradation via the 26S proteasome (Yu et al., 2007; Weidler et al., 2012; Chen et al., 2015, 2021; Liu et al., 2016, 2022; Miao et al., 2022). Photoactivated CRYs interact with over 80 proteins, including transcription factors, chromatin remodeling factors, RNA binding proteins, E3 ligases, and so on, to transduce blue-light signals in plants (Wang and Lin, 2020; Qu et al., 2024). These diverse interactions highlight the complexity and importance of CRY-mediated signal transduction. Plant CRYs play pivotal roles in regulating various developmental processes throughout the entire plant life cycle, especially in promoting plant photomorphogenesis (Ahmad and Cashmore, 1993; Lin et al., 1998) and photoperiod-dependent floral initiation (Guo et al., 1998; El-Din El-Assal et al., 2001; Zhao et al., 2022).

Temperature is another crucial environmental cue that affects plant growth and development. Different plant species have specific minimum and maximum temperature ranges they can tolerate. Within these limits, moderate increases in temperature (also referred to as warm or high ambient temperatures) can trigger thermomorphogenesis, a process that modifies plant growth and development. However, environmental temperatures that exceed a plant’s tolerance limits can induce cold-stress or heat-stress responses, leading to plant damage. Plants have evolved sophisticated mechanisms to deal with the adverse effects of cold and heat stress, enabling their survival under these stressful conditions. The heat-shock transcription factors (HSFs) and heat-shock proteins (HSPs) play dominant roles in activating heat-shock responses. HSFs recognize a consensus heat-shock element in the promoters of *HSPs* and regulate their expression in response to stressful high temperatures (Kotak et al., 2007; von Koskull-Döring et al., 2007). HSPs act as molecular chaperones to prevent or reverse the inactivation and irreversible aggregation of proteins under heat-stress conditions (Baniwal et al., 2007; Basha et al., 2012; Doyle et al., 2013; Kotak et al., 2007; Schramm et al., 2008; Vierling, 1991; von Koskull-Döring et al., 2007).

Photoreceptors and light signaling molecules are known to regulate plant thermal sensing and thermal responses. For instance, the red-light receptor PHYTOCHROME B (phyB) functions as a thermal sensor (Jung et al., 2016; Legris et al., 2016; Chen et al., 2022). Elevated ambient temperatures inactivate phyB by facilitating reversion of phyB from the active Pfr form to the inactive Pr form (Jung et al., 2016; Legris et al., 2016). Light-activated phyB has been shown to participate in the regulation of both basal (under heat-shock conditions) and acquired plant thermotolerance (under prolonged heat-shock conditions) (Song et al., 2017; Arico et al., 2019; Han et al., 2019). CRYs have previously been shown to regulate plant cold acclimation and thermomorphogenesis under ambient temperature conditions (Ma et al., 2016; Li et al., 2021). For example, photoactivated CRY1 inhibits plant thermomorphogenesis by interacting with PHYTOCHROME-INTERACTING FACTOR 4 (PIF4) to repress its transcriptional activation activity (Ma et al., 2016). PIF4 is a master regulator that integrates the blue-light and red-light signaling pathways, promoting gene expression for plant thermosensory responses under warm temperatures (28°C) (Koini et al., 2009; Kumar et al., 2012). CRY1 also interacts with TEOSINTE BRANCHED 1/CYCLOIDEA/PCF 17 (TCP17) to indirectly regulate PIF4 activity (Zhou et al., 2019). Low ambient temperatures (16°C) promote the degradation of CRY2 (Ma et al., 2021), whereas cold (or stressfully low) temperatures (4°C) stabilize phosphorylated CRY2 (Li et al., 2021), enhancing its interaction with CONSTITUTIVE PHOTOMORPHOGENIC 1 (COP1). The CRY2–COP1 interaction results in the suppression of ELONGATED HYPOCOTYL 5 (HY5) degradation, promoting the expression of freezing-tolerance genes and plant cold acclimation (Li et al., 2021). CRY2 also regulates plant thermosensory flowering at low ambient temperatures by interacting with CRY2 INTERACTING SPLICING FACTOR 1 (CIS1) to inhibit the accumulation of the FLOWERING LOCUS M beta (FLM-β) splice variant (Pose et al., 2013; Zhao et al., 2022). A recent study has shown that CRY1 may promote plant thermotolerance by promoting the nuclear localization of HEAT SHOCK TRANSCRIPTION FACTOR A1d (HSFA1d) (Gao et al., 2023), coupling CRY-mediated light signaling with plant heat-stress responses. COP1 and HY5, two key regulators of plant photomorphogenesis, have also been reported to participate in both plant cold acclimation and thermomorphogenesis. Elevated temperatures induce the nuclear import of COP1, leading to the excessive degradation of HY5 at higher ambient temperatures (Park et al., 2017). Conversely, cold temperatures trigger the nuclear depletion of COP1, thereby stabilizing HY5 (Catalá et al., 2011; Li et al., 2021).

In this study, we found that CRY1 negatively regulates plant thermotolerance by decreasing its interaction with COP1 in response to heat stress, leading to enhanced degradation of HY5 and suppression of *HSF* expression. Our study reveals a novel mechanism by which CRY1-mediated blue-light signaling suppresses plant thermotolerance, providing insights into how CRY1 integrates blue-light and temperature signals to affect plant growth and development. Together, our study and previous studies highlight the multifaceted functions of the CRY–COP1–HY5 module in light, cold-stress, and heat-stress signaling in *Arabidopsis*.

## Results

### CRY1 mediates blue-light suppression of thermotolerance in *Arabidopsis*

**Figure 1:**
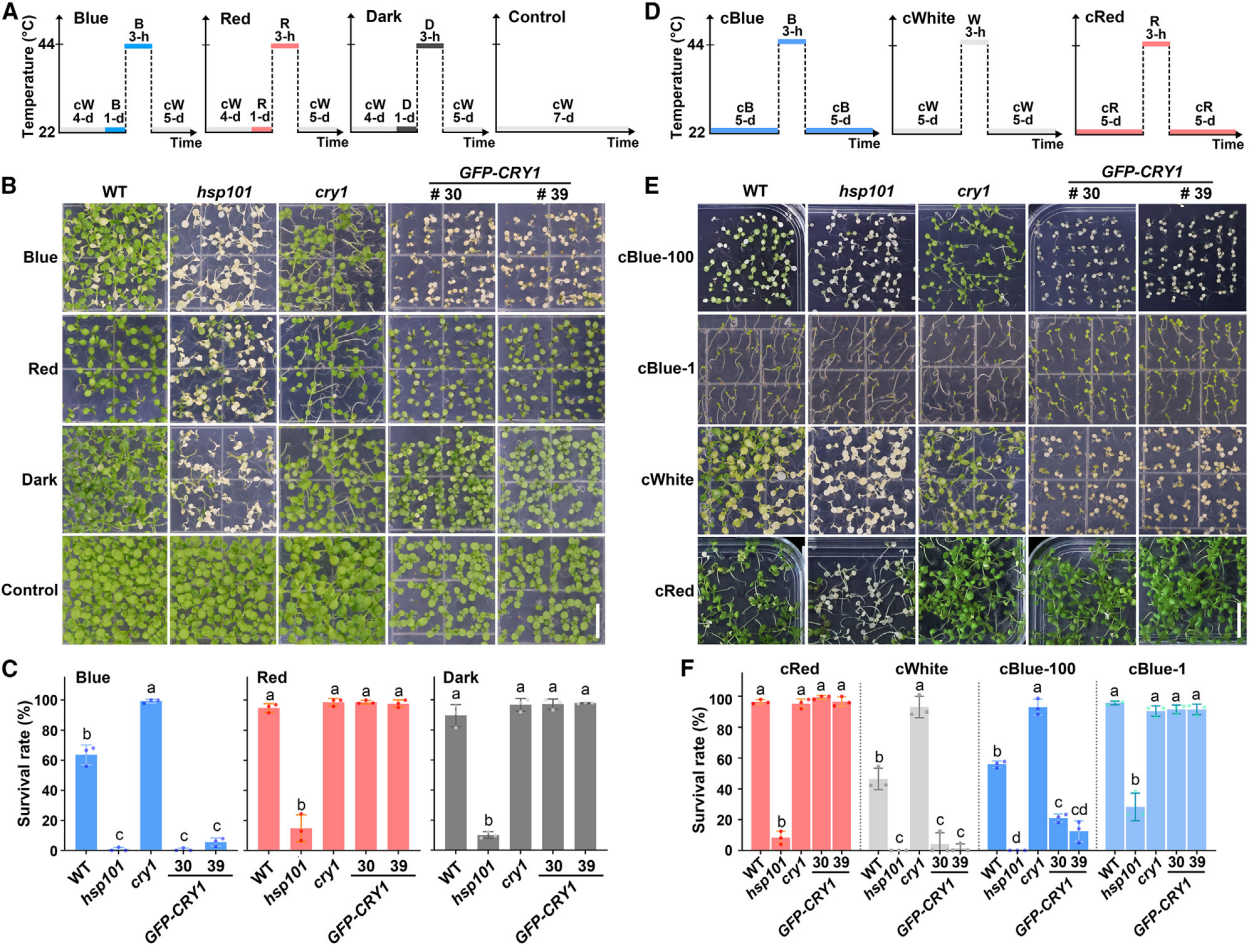
CRY1 mediates blue-light suppression of thermotolerance in *Arabidopsis*. **(A)** Representative charts showing different sets of heat-stress experimental conditions. Plants were initially grown on MS plates at 22°C in continuous white light (cW, 100 μmol m^−2^ s^−1^) for 4 days. Next, they were pretreated with blue light (B, 100 μmol m^−2^ s^−1^), red light (R, 100 μmol m^−2^ s^−1^), or darkness (D) for 1 day at 22°C before being subjected to heat-stress treatment at 44°C for 3 h. After the heat-stress treatment, the seedlings were allowed to recover for 5 days at 22°C under continuous white light (100 μmol m^−2^ s^−1^). **(B)** Representative thermotolerance phenotypes of the indicated genotypes, with *hsp101* seedlings serving as negative controls. Lines #30 and #39 are two independent *GFP-CRY1* transgenic lines. Scale bar, 1 cm. **(C)** Quantification of the survival rates of the seedlings in **(B)**. **(D)** Representative charts showing the heat-stress experiments under continuous-light conditions. Plants were initially grown on MS plates at 22°C under different light conditions, continuous blue light (1 μmol m^−2^ s^−1^ and 100 μmol m^−2^ s^−1^), continuous white light (100 μmol m^−2^ s^−1^), or continuous red light (100 μmol m^−2^ s^−1^), for 5 days. They were then subjected to a heat-stress treatment at 44°C for 3 h at day 5, followed by a recovery period of 5 days at 22°C under the respective light conditions. **(E)** Representative thermotolerance phenotypes of the indicated genotypes under continuous-light conditions. cBlue-100, continuous 100 μmol m^−2^ s^−1^ of blue light; cBlue-1, continuous 1 μmol m^−2^ s^−1^ of blue light; cWhite, continuous white light (100 μmol m^−2^ s^−1^); and cRed, continuous red light (100 μmol m^−2^ s^−1^). Scale bar, 1 cm. **(F)** Quantification of the survival rates of the seedlings in **(E)**. For **(C)** and **(F)**, the data are presented as the mean ± SD of three biological replicates, with approximately 50 plants per genotype examined in each biological replicate. Different letters indicate statistically significant differences in survival rates between genotypes within each treatment (one-way ANOVA followed by Tukey’s multiple comparison test, *p* < 0.05).

We initially used a set of experimental conditions to study the role of blue light and CRYs in *Arabidopsis* thermotolerance (Figure 1A–1C). To eliminate the gating effect of the circadian clock and minimize the morphological variations among plants, we grew seedlings in continuous white light for 4 days at 22°C, referred to as the basal growth condition, to ensure that they reached a similar developmental stage. Plants left in the basal growth condition throughout the experiments served as the controls. We then treated the seedlings with blue light, red light, or darkness for 1 day at 22°C, referred to as pretreatment conditions, before subjecting them to heat-shock treatment at 44°C for 3 h under the same light conditions as the pretreatment. After the heat shock, the seedlings were allowed to recover at 22°C for 5 days under continuous white light, referred to as the recovery condition. We then quantified the survival rate after the 5-day recovery period, which was defined as the percentage of healthy seedlings at the end of the recovery period, and used this survival rate as an indicator of thermotolerance. As expected, *hsp101* mutant seedlings, which lack the molecular chaperone HSP101, were hypersensitive to heat stress under both dark and light pretreatment conditions (Figure 1B and 1C), confirming the effectiveness of our heat-stress treatment. Interestingly, the survival rates of wild-type (WT) seedlings pretreated with darkness and red light were higher than those of seedlings pretreated with blue light (Figure 1B and 1C), indicating that blue-light pretreatment may suppress thermotolerance. Consistent with this finding, the survival rates of *cry1* mutants were significantly higher than those of WT seedlings pretreated with blue light, whereas the survival rates of seedlings overexpressing *GFP-CRY1* were markedly lower than those of the WT (Figure 1B and 1C). We next tested whether blue-light inhibition of thermotolerance was dependent on light intensity by pretreating seedlings under different intensities of blue and red light (Supplemental Figure 1A). WT seedlings pretreated with different intensities of blue light exhibited a fluence-dependent suppression of thermotolerance, with a significantly higher survival rate under low blue light (Supplemental Figure 1B and 1C). By contrast, *GFP-CRY1* seedlings were more sensitive to heat stress, whereas *cry1* mutants were more resistant (Supplemental Figure 1B and 1C). However, WT, *cry1*, and *GFP-CRY1* seedlings pretreated with varying intensities of red light showed similar thermotolerance across all fluences of red light (Supplemental Figure 1B and 1C). These results suggest that CRY1 may mediate blue-light suppression of thermotolerance in *Arabidopsis*.

To verify the possible blue-light effect on thermotolerance, we examined thermotolerance by changing the basal growth condition from continuous white light to continuous red light but kept the other conditions unchanged (Supplemental Figure 2A). Under this condition, only blue-light-pretreated *cry1* and *GFP-CRY1* seedlings showed thermotolerance phenotypes (Supplemental Figure 2B and 2C). Because *cry1* and *GFP-CRY1* seedlings exhibit the same photomorphogenic phenotype as the WT in basal-growth red light (Gao et al., 2022), these findings also suggest that CRY1 mediates this suppression independently of seedling architecture. To further confirm our observation, we assessed the thermotolerance of seedlings by changing the basal, pretreatment, and recovery light conditions to continuous blue light, continuous white light, or continuous red light, while keeping the other conditions unchanged (Figure 1D). Again, we observed results similar to those obtained in other conditions (Figure 1E and 1F). After 3 h of heat shock, the WT, *cry1*, and *GFP-CRY1* seedlings grown under 1 μmol m^−2^ s^−1^ blue light displayed thermotolerance comparable to that of seedlings grown under red light (Figure 1E and 1F). The *cry1* mutants grown under both continuous white and strong blue light were resistant to heat stress, with survival rates much higher than that of the WT (Figure 1E and 1F). By contrast, plants overexpressing *GFP-CRY1* were heat sensitive under continuous white-light and strong blue-light conditions, with significantly lower survival rates than the WT (Figure 1E and 1F). Seedlings in continuous red light demonstrated greater resistance to heat stress compared with those in blue light. For instance, WT seedlings in red light required a longer heat-shock period (5.5 h) to exhibit visible signs of heat-shock damage (Supplemental Figure 3). *cry1* and *GFP-CRY1* seedlings showed thermotolerance similar to that of the WT in continuous red light (Supplemental Figure 3). These results provide further evidence that CRY1 mediates the blue-light suppression of thermotolerance under our experimental conditions.

**Figure 2:**
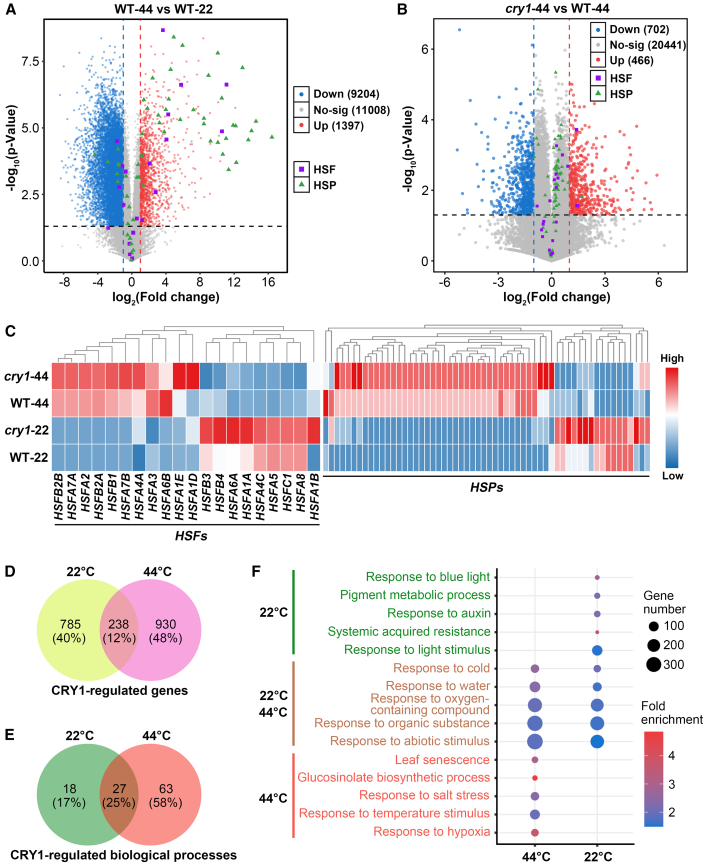
CRY1 regulates the expression of heat-stress-responsive genes. **(A)** Differentially expressed genes in WT plants in response to heat stress. **(B)** CRY1-regulated genes in response to heat stress. In **(A)** and **(B)**, the expression of *HSFs* and *HSPs* is highlighted. Differentially expressed genes are defined as |log_2_(fold change)| ≥ 1, *p* < 0.05. Down, downregulated genes; No-sig, genes with no significant expression change; Up, upregulated genes. **(C)** Heatmaps of RNA-sequencing transcriptome analysis showing the expression of *HSF* or *HSP* genes in both WT and *cry1*. Genes with log_2_(FPKM) values less than −5 in all four samples (considered to be low-expressed genes) have been omitted from the original list. Twenty detected *HSFs* and 58 detected *HSPs* are shown in the heatmaps. **(D)** A Venn diagram depicting the number of CRY1-regulated genes at 22°C or 44°C. Plants were grown on MS plates at 22°C in continuous white light (100 μmol m^−2^ s^−1^) for 4 days and then acclimated in blue light (100 μmol m^−2^ s^−1^) for 1 day before heat shock. RNA-sequencing samples were collected after heat-stress treatment at 44°C for 2 h under blue light (referred to as 44°C) or collected at 22°C under blue light (referred to as 22°C). **(E)** GO analyses showing the number of biological processes regulated by CRY1 at 22°C or 44°C. **(F)** Five representative enriched biological processes that were either co-regulated by CRY1 at both 22°C and 44°C or independently regulated by CRY1 at 22°C or 44°C.

To investigate the molecular mechanism underlying CRY1-mediated blue-light suppression of thermotolerance, we first performed transcriptome profiling of seedlings with basal growth in white light and pretreated with blue light without heat shock (referred to as 22°C) or with heat-shock treatment for 2 h (referred to as 44°C) (Supplemental Figure 4). As expected, most of the *HSFs* and their target *HSPs* were highly induced upon heat-shock treatment in WT seedlings (Figure 2A; Supplemental Dataset 1). The transcript levels of most *HSFs* and *HSPs* were upregulated in *cry1* mutants compared with WT plants in response to heat stress (Figure 2B and 2C). Although the induction of most *HSFs* and *HSPs* by CRY1 is less than two-fold, they show increased expression in *cry1* mutants (Figure 2B and 2C), with some *HSFs* and *HSPs* being upregulated even at normal temperature in the *cry1* mutants (Figure 2C; Supplemental Figure 5A; Supplemental Dataset 2), consistent with CRY1 acting as a negative regulator of thermotolerance. We performed both Gene Ontology (GO) analysis and Gene Set Enrichment Analysis (GSEA) (Subramanian et al., 2005; Powers et al., 2018; Reimand et al., 2019; Vocale et al., 2021) of the heat-regulated transcriptome. Both analyses showed significant enrichment of pathways involved in responses to heat stress and oxidative stress (Supplemental Figure 5B–5D; Supplemental Datasets 3 and 4). Interestingly, many light-responsive genes were also heat responsive (Supplemental Figure 5B–5D; Supplemental Datasets 3 and 4), consistent with an important role for light signaling in the heat-shock response. We defined all the genes regulated by CRY1 under normal (22°C) and heat-stress (44°C) conditions as CRY1-regulated genes (Figure 2B; Supplemental Figure 5A; Supplemental Datasets 5 and 6). Interestingly, only 12% of the CRY1-regulated genes were shared by normal and heat-stress conditions (Figure 2D). GO analysis revealed that the CRY1-regulated genes specific to normal or heat-stress conditions were enriched in distinct biological processes (Figure 2E and 2F; Supplemental Datasets 7 and 8). Under the normal temperature condition, a number of the most highly enriched terms in the CRY1-regulated genes were associated with light responses, whereas under heat stress, a number were associated with responses to temperatureand other stresses (Figure 2F). These findings are consistent with the hypothesis that CRY1-mediated light signaling regulates plant heat-shock response and thermotolerance.

### Heat stress represses the blue-light-dependent phosphorylation of CRY1

We next sought to understand how heat stress affects the CRY1 photoreceptor itself. CRY1 is known to undergo blue-light-dependent oligomerization, phosphorylation, ubiquitination, and degradation (Wang et al., 2016; Liu et al., 2017, 2022; Chen et al., 2021; Miao et al., 2022). We first examined blue-light-dependent CRY1 oligomerization under normal and heat-stress conditions by co-immunoprecipitation (co-IP) using plants co-expressing FGFP-CRY1 (CRY1 fused to a FLAG tag and a green fluorescent protein) and Myc-CRY1 (Liu et al., 2020). Blue-light-specific CRY1 oligomerization was observed under both conditions (Supplemental Figure 6A). CRY1 is distributed in both the nucleus and cytoplasm, and photoactivated nuclear CRY1 is known to oligomerize into photobodies (Liu et al., 2022). We therefore explored the effect of heat shock on blue-light-induced CRY1 photobody formation. Consistent with our co-IP findings in plants (Supplemental Figure 6A), heat shock did not disrupt the formation of nuclear CRY1 photobodies (Supplemental Figure 6B and 6C). It appears that heat stress does not significantly affect the photo-oligomerization or photobody formation activities of the CRY1 photoreceptor.

**Figure 3:**
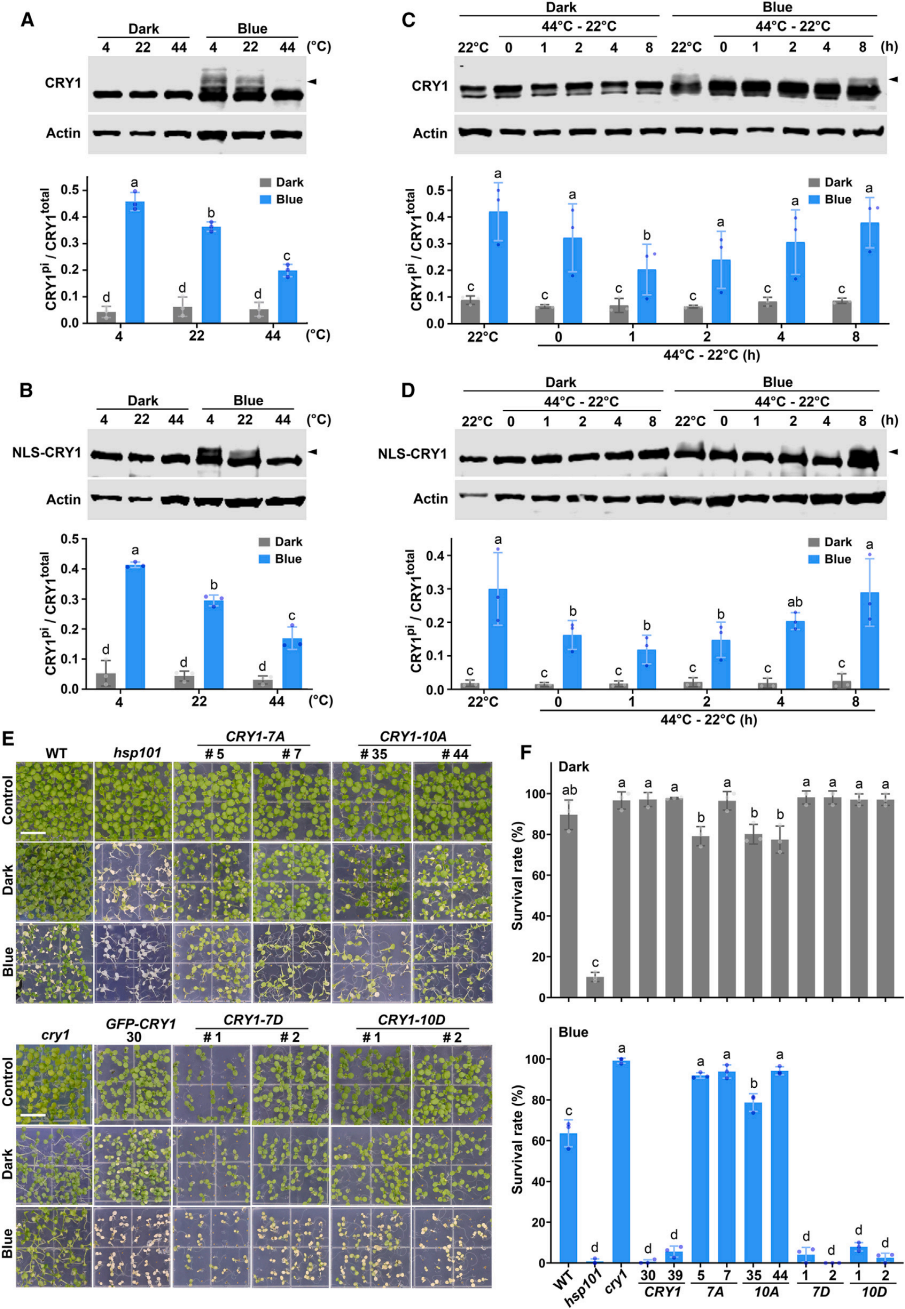
Heat stress represses the blue-light-dependent phosphorylation of CRY1. **(A and B)** Five-day-old wild-type **(A)** or *NLS-GFP-CRY1* overexpression **(B)** seedlings grown at 22°C under constant darkness or blue light (100 μmol m^−2^ s^−1^) were treated at 4°C, 22°C, or 44°C for 4 h under the same light conditions. **(C and D)** Five-day-old wild-type **(C)** or *NLS-GFP-CRY1* overexpression **(D)** seedlings grown under constant darkness or blue light (100 μmol m^−2^ s^−1^) were subjected to a 1-h treatment at 44°C and then recovered at 22°C for the indicated time under the same light conditions. For **(A)–(D)**, the levels of CRY1 and Actin were detected using anti-CRY1 and anti-Actin 2 antibodies, respectively, with Actin serving as the loading control. Arrowheads indicate phosphorylated CRY1. The degree of CRY1 phosphorylation was determined by normalizing the amount of phosphorylated CRY1 to that of total CRY1 (CRY1^pi^/CRY1^total^) and is presented as the mean ± SD (*n* = 3 individual immunoblots). Different letters indicate statistically significant differences between samples under darkness and blue light (two-way ANOVA followed by Sidak’s multiple comparison test, *p* < 0.05). **(E)** Representative thermotolerance phenotypes of the indicated genotypes. The experimental procedures and treatments were the same as those in Figure 1A. Pound signs followed by a number indicate different transgenic lines. Scale bars, 1 cm. **(F)** Quantification of the survival rates of the seedlings in **(E)**. The data are presented as the mean ± SD of three biological replicates, with approximately 50 plants per genotype examined in each biological replicate. Different letters indicate statistically significant differences in survival rates between genotypes (one-way ANOVA followed by Tukey’s multiple comparison test, *p* < 0.05).

We then examined the blue-light-dependent phosphorylation of CRY1 in response to heat stress. Consistent with a previous report (Li et al., 2021), the level of phosphorylated CRY1, which exhibits a retarded migration band that can be eliminated by a phosphatase (λ-PPase), was higher at 4°C than at 22°C (Figure 3A; Supplemental Figure 7A and 7B). By contrast, the level of phosphorylated CRY1 decreased in response to 44°C heat-shock treatment (Figure 3A; Supplemental Figure 7C and 7D). Nuclear CRY1 (NLS-CRY1) was also found to dephosphorylate at 44°C and to exhibit enhanced phosphorylation at 4°C under continuous blue light (Figure 3B; Supplemental Figure 7E and 7F). During recovery from heat stress, phosphorylated CRY1 gradually began to reaccumulate (Figure 3C and 3D). Phosphorylated CRY1 increased in both WT and *NLS-CRY1* transgenic plants after heat-treated (44°C) plants were transferred to 22°C for 8 h (Figure 3C and 3D). Consistent with previous reports that phosphorylation is required for CRY1 degradation, the CRY1 protein became more stable during heat-shock treatments. Markedly more NLS-CRY1 protein was detected at 44°C than at 22°C when plants were transferred from darkness to blue light (Supplemental Figure 8). We next assessed the functions of CRY1 phosphorylation in plant thermotolerance. Transgenic plants expressing the non-phosphorylatable CRY1 mutants CRY1-7A or CRY1-10A (Gao et al., 2022) exhibited similar higher-thermotolerance phenotypes compared with the *cry1* mutant plants, whereas transgenic plants expressing the phosphomimetic CRY1 mutants CRY1-7D or CRY1-10D (Gao et al., 2022) were hypersensitive to heat stress in blue light (Figure 3E and 3F; Supplemental Figure 9). These results collectively demonstrate that the blue-light-induced phosphorylation of CRY1 is intrinsic to its activity in the suppression of thermotolerance.

**Figure 4:**
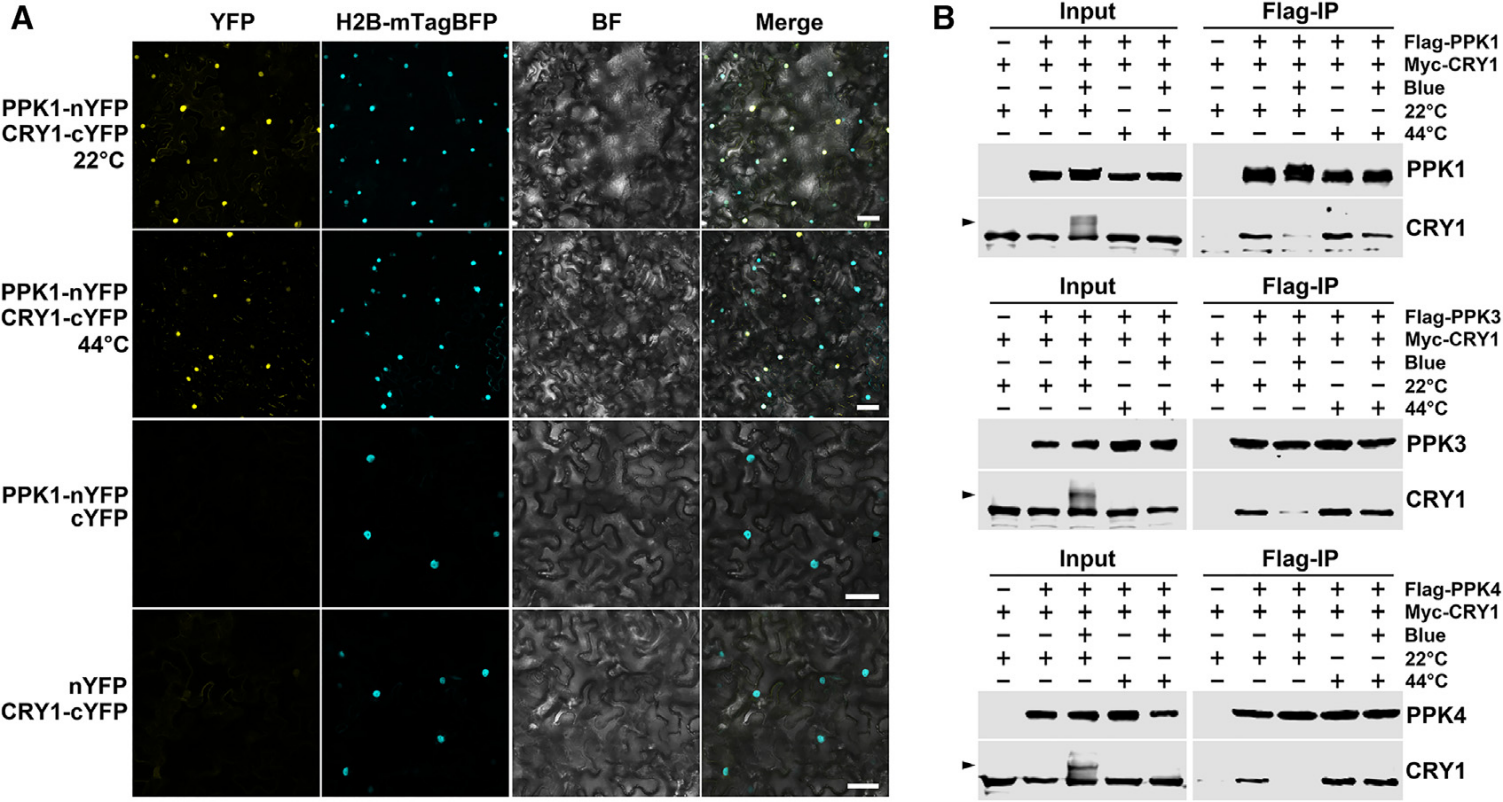
Heat stress does not inhibit the direct interaction between CRY1 and PPKs but may suppress the kinase activity of PPKs. **(A)** Confocal images showing the reconstituted fluorescence of CRY1 and PPK1 in BiFC assays. BiFC transient expression experiments were performed in tobacco by co-injecting *Agrobacterium* strains carrying the indicated nYFP and cYFP pairs, along with H2B-mTagBFP as a nuclear marker. Fluorescence signals were assessed 72 h post-transfection by confocal microscopy. The plants were subjected to treatments at 22°C or 44°C for 1 h prior to imaging. BF, bright field; scale bars, 50 μm. **(B)** Co-IP results demonstrating the interactions between PPKs and CRY1 in HEK293T cells. Cells co-expressing *Myc-CRY1* and *Flag-PPKs* (*PPK1*/*PPK3*/*PPK4*) were kept in the dark (− blue) or exposed to blue light (100 μmol m^−2^ s^−1^, + blue) for 1 h at 22°C or 44°C. Immunoprecipitations were performed using FLAG-conjugated beads. The levels of PPKs and CRY1 were detected using anti-FLAG and anti-CRY1 antibodies, respectively. Arrowheads indicate phosphorylated CRY1.

We suspected that heat stress might regulate CRY1 phosphorylation by disrupting its interaction with its kinases. We have previously reported that PPKs (PPK1 to PPK4) phosphorylate CRY1 in blue light (Gao et al., 2022). We therefore investigated the direct physical interaction between CRY1 and PPK1 under normal and heat-stress conditions using a bimolecular fluorescence complementation (BiFC) assay and a co-IP assay. Strong BiFC signals resulting from the interaction between PPK1-nYFP and CRY1-cYFP were detected in the nucleus at both 22°C and 44°C in tobacco leaves (Figure 4A). Co-IP assays in human embryo kidney 293T (HEK293T) cells further confirmed a direct interaction between CRY1 and PPKs at both 22°C and 44°C (Figure 4B). PPKs exhibited a higher affinity for non-phosphorylated CRY1 (Figure 4B), as reported previously (Gao et al., 2022). However, despite the interactions between PPKs and CRY1 at both temperatures, almost no slow-migrating phosphorylated CRY1 band was detected at 44°C in the presence of PPKs under blue light (Figure 4B), indicating that PPKs are inactive at phosphorylating CRY1 at high temperatures or that other phosphatases dephosphorylate CRY1 at high temperatures. However, the detailed mechanisms involved require further investigation.

### Heat stress disrupts the interaction between CRY1 and COP1, leading to excessive degradation of HY5 under heat stress in blue light

**Figure 5:**
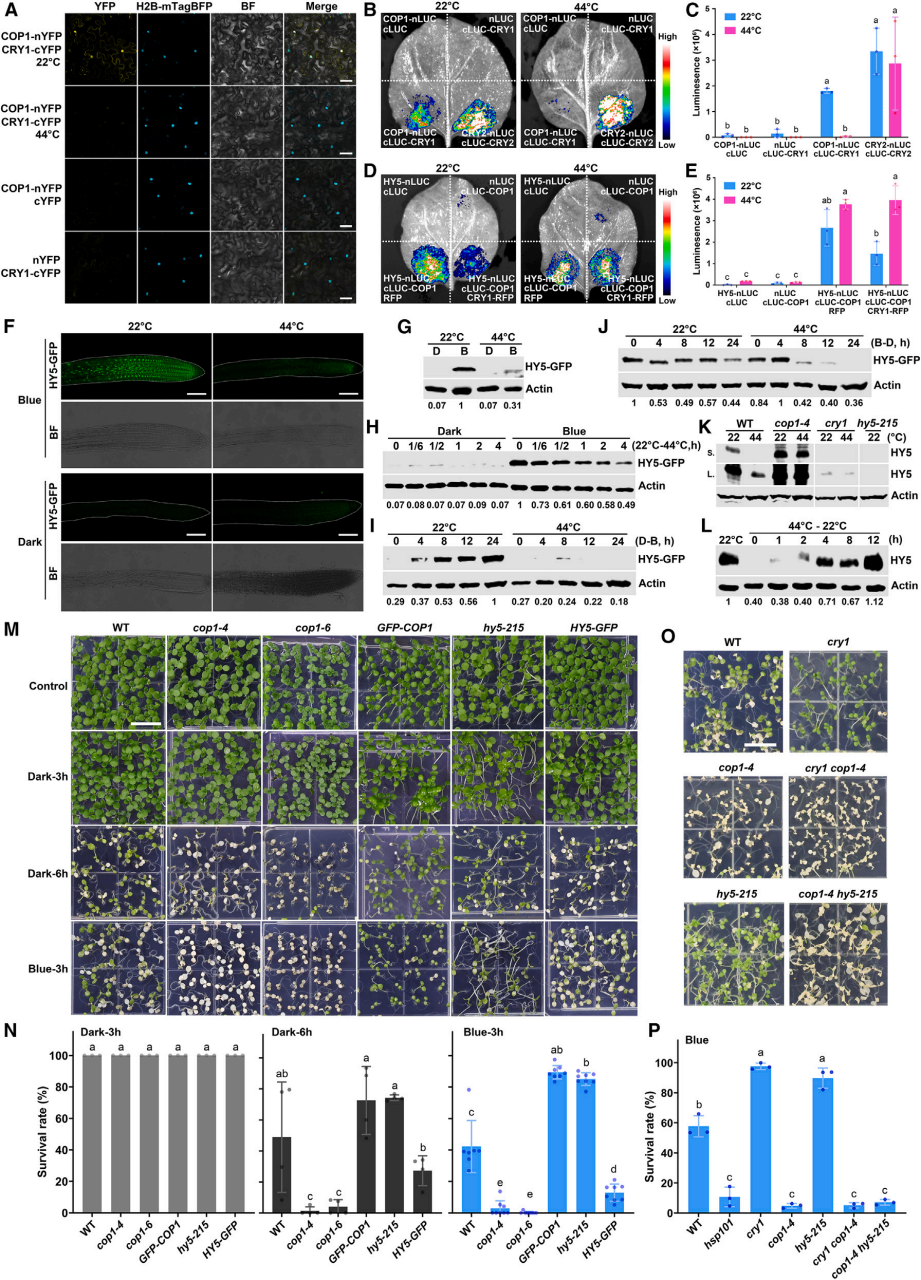
Heat stress disrupts the interaction between CRY1 and COP1, leading to excessive degradation of HY5 in response to heat stress in blue light. **(A and B)** BiFC assays **(A)** and split-LUC assays **(B)** demonstrating disruption of the COP1 and CRY1 interaction by heat stress. The experimental procedures and treatments were the same as those in Figure 3A. Fluorescence signals were assessed 72 h post-transfection using a confocal microscope, and luciferase activities were evaluated using a CCD camera. The plants were subjected to treatments at 22°C or 44°C for 1 h prior to imaging. BF, bright field; scale bars, 50 μm. **(C)** Quantification of the reconstituted luciferase activities shown in **(B)**. The data are presented as the mean ± SD of three independent replicates. Different letters indicate statistically significant differences (*p* < 0.05) determined by one-way ANOVA. **(D)** Split-LUC assays showing the interaction between COP1 and HY5 at 22°C and 44°C. The plants were subjected to treatments at 22°C or 44°C for 1 h prior to imaging. **(E)** Quantification of the reconstituted luciferase activities shown in **(D)**. The data were processed as described in **(C)**. **(F)** Confocal images showing reduced HY5 GFP fluorescence in response to heat stress. Four-day-old *proHY5::HY5-GFP* seedlings were grown in either continuous darkness or blue light (100 μmol m^−2^ s^−1^) at 22°C and then subjected to heat-stress treatment at 44°C for 4 h, followed by fixation in 4% paraformaldehyde before imaging. White dashes outline the roots. Scale bars, 100 μm. **(G)** Immunoblots showing the HY5-GFP protein levels extracted from the seedlings in **(F)**. **(H)** Immunoblots illustrating the degradation of HY5 in response to heat stress. Five-day-old HY5-GFP seedlings, grown under darkness or blue light (100 μmol m^−2^ s^−1^) at 22°C, were subjected to 44°C treatment for the indicated time under the same light conditions. **(I)** Immunoblots showing the inhibition of HY5 accumulation by heat stress in blue light. Five-day-old *HY5-GFP* seedlings, grown in constant darkness at 22°C, were transferred to blue light (100 μmol m^−2^ s^−1^) at 22°C or 44°C for the indicated time. **(J)** Immunoblots showing the accelerated degradation of HY5 under heat stress in the dark. Five-day-old *HY5-GFP* seedlings, grown under blue light (100 μmol m^−2^ s^−1^) at 22°C, were transferred to darkness at 22°C or 44°C for the indicated time. **(K)** Immunoblots showing endogenous HY5 protein levels in the indicated genotypes. Five-day-old seedlings grown at 22°C under blue light were treated at 22°C or 44°C for 4 h in blue light before protein extraction. Proteins from different genotypes were loaded on the same gel and presented in the same blot exposure. S, short exposure; L, long exposure. **(L)** Immunoblots demonstrating the re-accumulation of endogenous HY5 during recovery from heat stress. Five-day-old wild-type seedlings, grown under blue light (100 μmol m^−2^ s^−1^), were treated at 44°C for 4 h and then recovered at 22°C for the indicated time. **(M)** Representative thermotolerance phenotypes of the indicated genotypes are shown. The experimental procedures and treatments were the same as those in Figure 1A, except that the heat stress treatment of the dark-pretreated seedlings was prolonged to 6 h (Dark-6h). Scale bar, 1 cm. **(N)** Quantification of the survival rates of the seedlings in **(M)**. **(O)** Representative thermotolerance phenotypes of the indicated genotypes pretreated in blue light. The experimental procedures were the same as those in Figure 1A. Scale bar, 1 cm. **(P)** Quantification of the survival rates of seedlings in **(O)**. In **(G)–(J)**, the levels of HY5 and Actin were detected using anti-GFP and anti-Actin antibodies, respectively, with Actin serving as the loading control. In **(K)** and **(L)**, the levels of HY5 and Actin were detected using anti-HY5 and anti-Actin antibodies, respectively. In **(G)–(J)** and **(L)**, the relative intensities of HY5 protein are shown below the immunoblots, normalized to the intensities of Actin, and the highest intensity of HY5 on a given blot was set to 1. In **(N)** and **(P)**, the data are presented as the mean ± SD of three biological replicates, with approximately 50 plants per genotype examined in each biological replicate. Different letters indicate statistically significant differences in survival rates between genotypes within each treatment (one-way ANOVA followed by Tukey’s multiple comparison test, *p* < 0.05).

Phosphorylation of CRY1 was previously reported to increase its affinity for COP1 (Gao et al., 2022), and the CRY–COP1–HY5 axis, involving CRY-dependent inhibition of COP1 and accumulation of HY5, leads to plant responses to cold and warm ambient temperatures (Catalá et al., 2011; Delker et al., 2014; Toledo-Ortiz et al., 2014; Gangappa and Kumar, 2017; Park et al., 2017; Li et al., 2021; Kim et al., 2022). We postulated that CRY1 might also regulate plant thermotolerance through the CRY–COP1–HY5 axis. To test this hypothesis, we examined the interaction between CRY1 and COP1 at 22°C and 44°C. The BiFC signal resulting from the interaction between COP1-nYFP and CRY1-cYFP was detected at 22°C, but no BiFC signal was detected at 44°C (Figure 5A). Consistent with this finding, the firefly luciferase (LUC) activity between COP1-nLUC and cLUC-CRY1 was reconstituted in a split-LUC complementation assay at 22°C, whereas much less LUC activity was observed at 44°C (Figure 5B and 5C). These results are consistent with the hypothesis that inactivation of CRY1 phosphorylation at 44°C decreases the CRY1–COP1 interaction.

CRYs interact with COP1 via their VP (Val-Pro) motifs to competitively inhibit the interaction between COP1 and its substrates, such as HY5, in blue light (Podolec and Ulm, 2018; Lau et al., 2019; Ponnu et al., 2019). We next investigated whether the decreased interaction between CRY1 and COP1 under heat stress resulted in an enhanced interaction between COP1 and HY5 in response to heat stress. As expected from the above hypothesis, CRY1-RFP inhibited the COP1–HY5 interaction, as indicated by the weakened LUC signal reconstituted by HY5-nLUC and cLUC-COP1 at 22°C but not at 44°C (Figure 5D and 5E). In other words, heat inhibits both CRY1 phosphorylation and CRY1–COP1 interaction, resulting in increased COP1–HY5 interaction in heat.

Because COP1 is the E3 ligase of HY5 (Osterlund et al., 2000), the above findings prompted us to postulate that HY5 protein may undergo excessive degradation under heat stress in the presence of blue light. To test this hypothesis, we first evaluated the fluorescence intensity of HY5-GFP driven by the *HY5* native promoter in response to high temperature. HY5-GFP fluorescence accumulated in blue light at 22°C, but the fluorescence signal of HY5-GFP decreased markedly after heat-stress treatment at 44°C (Figure 5F; Supplemental Figure 10), suggesting the degradation of HY5 protein in response to heat stress. By contrast, almost no HY5-GFP fluorescence was detected in darkness (Figure 5F; Supplemental Figure 10). This result was confirmed by immunoblotting analysis of the proteins extracted from these seedlings. Much less HY5-GFP protein was detected under blue light after heat-stress treatment at 44°C for 4 h (Figure 5G). The level of HY5 protein gradually decreased in response to heat stress under blue light (Figure 5H). We further analyzed the blue-light-dependent high-temperature-regulation of HY5 protein stability and found that heat stress inhibited HY5 protein accumulation in response to blue light (Figure 5I). HY5 exhibited substantial accumulation in response to blue light at 22°C, but its accumulation was markedly reduced at 44°C under the same light condition (Figure 5I). Consistent with this result, heat stress accelerated the degradation of HY5 in response to darkness. As shown in Figure 5J, HY5-GFP underwent considerably faster degradation at 44°C when plants were transitioned from blue light to darkness. To further investigate whether COP1 is responsible for the degradation of HY5 under heat stress, we assessed endogenous HY5 protein levels with an anti-HY5 antibody in WT, *cop1*, *cry1*, and *hy5* mutants under blue light. The *hy5* mutants were used as a negative control. As expected, more endogenous HY5 accumulated in the *cop1* mutants and less in the *cry1 mutants*at 44°C (Figure 5K). We also examined the polyubiquitination of HY5 protein in *cop1* mutants in response to heat stress using tandem ubiquitin binding entity 2 co-IP assays (Hjerpe et al., 2009; Chen et al., 2021; Liu et al., 2022). The polyubiquitination of HY5 protein increased in response to heat shock, but not in *cop1* mutants (Supplemental Figure 11), confirming that COP1 mediates the polyubiquitination and degradation of HY5 under heat stress. Consistent with this finding, levels of endogenous HY5 protein increased markedly during recovery from heat stress in the presence of blue light (Figure 5L). Taken together, these results demonstrate that, under blue light, heat stress plays a crucial role in regulating the stability of HY5 protein.

We next assessed the thermotolerance of *hy5* and *cop1* mutants, along with *GFP-COP1*- and *HY5-GFP*-overexpressing plants. The experimental treatments mirrored those shown in Figure 1, with the addition of a prolonged 6-h heat-stress treatment in darkness (Figure 5M and 5N) and a prolonged 5.5-h heat shock in red light (Supplemental Figure 12A). After 3 h of heat shock in darkness, no distinct thermotolerance phenotypes were observed among the different genotypes (Figure 5M and 5N). However, after 6 h of heat stress in darkness, the *cop1* mutants (*cop1-4* and *cop1-6*) exhibited hypersensitivity to heat stress, whereas the *hy5* mutant, *GFP-COP1*, and *HY5-GFP* plants showed thermotolerance similar to that of the WT (Figure 5M and 5N). By contrast, after 3 h of heat shock in blue light, both *cop1* mutants and *HY5-GFP*-overexpressing plants exhibited hypersensitivity to heat stress, whereas *GFP-COP1* and *hy5* mutants showed insensitivity compared with the WT (Figure 5M and 5N). Consistent with this finding, *cop1* mutants displayed hypersensitivity to heat stress when grown under continuous light, whereas *hy5* mutants showed insensitivity (Supplemental Figure 12). These results further reinforce the hypothesis that blue light suppresses thermotolerance in *Arabidopsis* and that the light signaling component COP1 enhances *Arabidopsis* thermotolerance, whereas HY5 diminishes it.

To explore the genetic interactions among CRY1, COP1, and HY5 in the regulation of plant heat-stress responses, we examined the thermotolerance of the *cry1cop1* and *cop1hy5* double mutants in the blue-light conditions described in Figure 1A. The *cry1cop1* double mutants exhibited heat-sensitive phenotypes similar to those of *cop1* mutants (Figure 5O and 5P). This observation that COP1 acts epistatically to CRY1 in the regulation of plant thermotolerance suggests that COP1 acts downstream of CRY1 to positively regulate plant heat tolerance. However, the *cop1hy5* double mutants also displayed a heat-sensitive phenotype in the presence of blue light, resembling that of the *cop1* single mutants (Figure 5O and 5P). This result indicates that COP1 is epistatic to HY5 with respect to thermotolerance in blue light. These findings imply that, in addition to regulating HY5 proteolysis in response to heat stress, COP1 may also modulate plant thermotolerance in an HY5-independent manner. These findings are consistent with previous studies suggesting that HY5 alone cannot fully explain the regulation of photomorphogenesis by COP1 (Chory, 1992; Ang and Deng, 1994; Gangappa and Kumar, 2017).

Together, our results demonstrate that, under blue-light conditions, heat stress disrupts the interaction between CRY1 and COP1, leading to an intensified interaction between COP1 and HY5 in response to heat stress and thus promoting degradation of HY5 under heat-stress conditions.

### HY5 binds to the promoters of *HSFs* to repress their transcription

**Figure 6:**
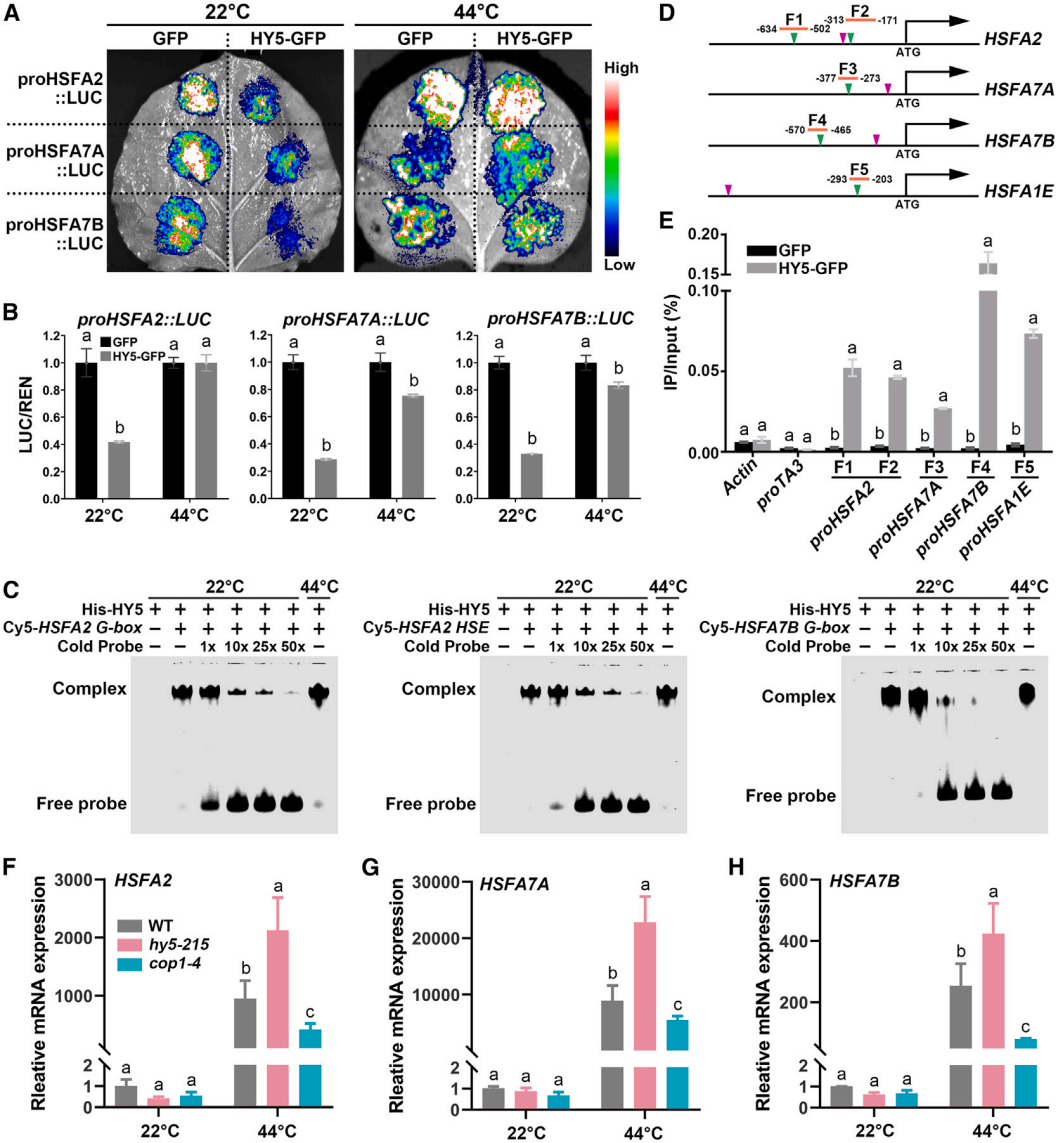
HY5 binds to the promoters of *HSFs* to repress their transcription. **(A)** Dual-LUC assays demonstrating the regulation of *HSF* transcription by HY5. *Agrobacterium* strains harboring effector and reporter plasmids were co-injected into tobacco leaves. Luciferase activities were evaluated using a CCD camera 72 h after transfection. The plants were subjected to treatments at 22°C or 44°C for 1 h before imaging. **(B)** Quantification of the relative *HSF* reporter activities in **(A)**. Relative LUC activities normalized to REN (*Renilla* luciferase) activities (LUC/REN) are shown. The data are presented as the mean ± SD of three independent replicates. The levels of LUC activity co-injected with GFP at 22°C and 44°C were set to 1. Different letters indicate statistically significant differences in LUC/REN between GFP and HY5-GFP samples at 22°C or 44°C (one-way ANOVA followed by Tukey’s multiple comparison test, *p* < 0.05). **(C)** EMSAs showing the binding of HY5 to the promoter DNA probes of *HSFA2* and *HSFA7B*. The promoter DNA probes were labeled with Cy5. Unlabeled promoter DNA probes are shown as cold probes. **(D)** Diagrams depicting the putative promoters of *HSFA2*, *HSFA7A*, *HSFA7B*, and *HSFA1E*. Green triangles indicate the G-box motifs, and purple triangles indicate the HSE motifs. F1, F2, F3, F4, and F5 indicate the fragments analyzed by qPCR in the ChIP assays. **(E)** Representative results of ChIP–qPCR assays showing the association of HY5 with the *HSFA2*, *HSFA7A*, *HSFA7B*, and *HSFA1E* promoters. *proHY5::HY5-GFP* and *pACT2::GFP* seedlings grown in blue light (100 μmol m^−2^ s^−1^) at 22°C for 10 days were harvested for ChIP–qPCR analysis using GFP-trap beads. The positions of F1, F2, F3, F4, and F5 are indicated in **(D)**. *Actin 2* and *TA3* promoters were used as negative controls. The data are shown as the mean ± SD (*n* = 3 technical replicates). Different letters indicate statistically significant differences in each gene between the GFP and HY5-GFP samples (one-way ANOVA followed by Tukey’s multiple comparison test, *p* < 0.05). **(F–H)** RT–qPCR analysis of *HSFA2*, *HSFA7A*, and *HSFA7B* expression in *hy5-215* and *cop1-4* in response to heat stress. The growth conditions were as shown in Figure 1A. Samples were collected after 1 h of heat shock treatment at 44°C or 22°C under blue light. Gene expression levels were normalized to that of *Actin 2*. The relative expression levels of the genes in the WT at 22°C were set to 1. The data are presented as the mean ± SD of three independent replicates. Different letters indicate statistically significant differences between genotypes within each treatment (one-way ANOVA followed by Tukey’s multiple comparison test, *p* < 0.05).

HSFs are highly conserved central regulators of plant heat-stress responses, and they play crucial roles in activating heat-shock responses. HY5 was reported to bind to the promoter of *HSFA2* to repress its transcription, thus helping to regulate plant salinity tolerance (Yang et al., 2023). We hypothesized that HY5 might bind to the promoters of *HSFs* to regulate their expression and, consequently, modulate plant thermotolerance under blue light. To test this hypothesis, we analyzed previously published HY5 chromatin immunoprecipitation (ChIP) sequencing data and found that HY5 bound to the promoters of 14 of 21 *HSFs* in *Arabidopsis* (Supplemental Figure 13) (Burko et al., 2020). We observed that HY5 seemed to preferentially bind to the promoters of heat-induced *HSFs*. For example, HY5 associated with the promoters of all 11 *HSFs* whose expression was induced by heat stress, whereas HY5 bound to the promoters of only 2 of 8 *HSFs* whose expression was inhibited by heat stress (Figure 2C; Supplemental Figure 13). We arbitrarily chose three HY5-binding *HSFs*, *HSFA2*, *HSFA7A*, and *HSFA7B*, and examined their transcriptional regulation by HY5 in plants. We performed a dual-LUC transient expression assay by co-injecting *Agrobacterium* strains containing *proHSFs::LUC* reporter plasmids and either *HY5-GFP* or *GFP* control effector plasmids into *Nicotiana benthamiana* leaves (Supplemental Figure 14A). The results showed that, compared with the GFP control, HY5 suppressed the *proHSFs::LUC* reporter activities at 22°C but not at 44°C (Figure 6A and 6B). Much weaker LUC activity was detected in the reporter groups co-transfected with *HY5-GFP* at 22°C (Figure 6A and 6B). We confirmed that the reduced inhibition of *HSF* reporter activities at 44°C primarily resulted from the degradation of HY5-GFP protein under heat stress. At 44°C, little HY5-GFP protein was detected, whereas the mRNA abundance of *HY5-GFP* remained comparable to that at 22°C (Supplemental Figure 14B and 14C), suggesting that HY5-GFP protein was degraded at 44°C, consistent with the observations in *Arabidopsis* (Figure 5F–5K). HY5 did not inhibit reporter gene activities driven by the *HSFB3* and *HSFB4* promoters, which showed no HY5 binding in the ChIP sequencing data at either 22°C or 44°C (Supplemental Figure 15).

In addition, we examined HY5 binding to some *HSF* promoters using an electrophoretic mobility shift assay (EMSA) and ChIP quantitative PCR (ChIP–qPCR). HY5 bound directly to the G-box motifs in the *HSFA2* and *HSFA7B* promoters and the heat-stress responsive element (HSE) motif in the *HSFA2* promoter *in vitro* at both 22°C and 44°C (Figure 6C). Increasing amounts of unlabeled cold probes significantly reduced the binding of HY5 to Cy5-labeled probes, but heat-stress temperatures did not affect HY5’s DNA-binding activity (Figure 6C). This result suggests that heat stress primarily regulates the protein stability of HY5 rather than its DNA-binding activity. The association of HY5 with *HSF* promoters was also confirmed by ChIP–qPCR in transgenic *HY5-GFP Arabidopsis*. The chromatin regions containing G-box motifs in the *HSFA2*, *HSFA7A*, *HSFA7B*, and *HSFA1E* promoters were highly enriched in the immunoprecipitation products pulled down by the recombinant HY5 protein in transgenic *HY5-GFP* seedlings (Figure 6D and 6E). These results suggest that HY5 can directly bind to the promoters of *HSFs* to modulate their expression.

We next examined the transcript levels of *HSFA2*, *HSFA7A*, and *HSF7B* in blue light at 22°C and 44°C in various mutant backgrounds, including the WT and *hy5* and *cop1* mutants. Quantitative reverse transcriptase PCR results demonstrated that *HSFA2*, *HSFA7A*, and *HSF7B* abundance increased in response to heat shock in the *hy5* mutants but decreased in the *cop1* mutants (Figure 6F–6H), consistent with the observed thermotolerance phenotypes of these mutants (Figure 5M and 5N). These results collectively demonstrate that HY5 binds to the promoters of *HSFs* to repress their transcription under heat stress, thus inhibiting *Arabidopsis* thermotolerance.

## Discussion

**Figure 7:**
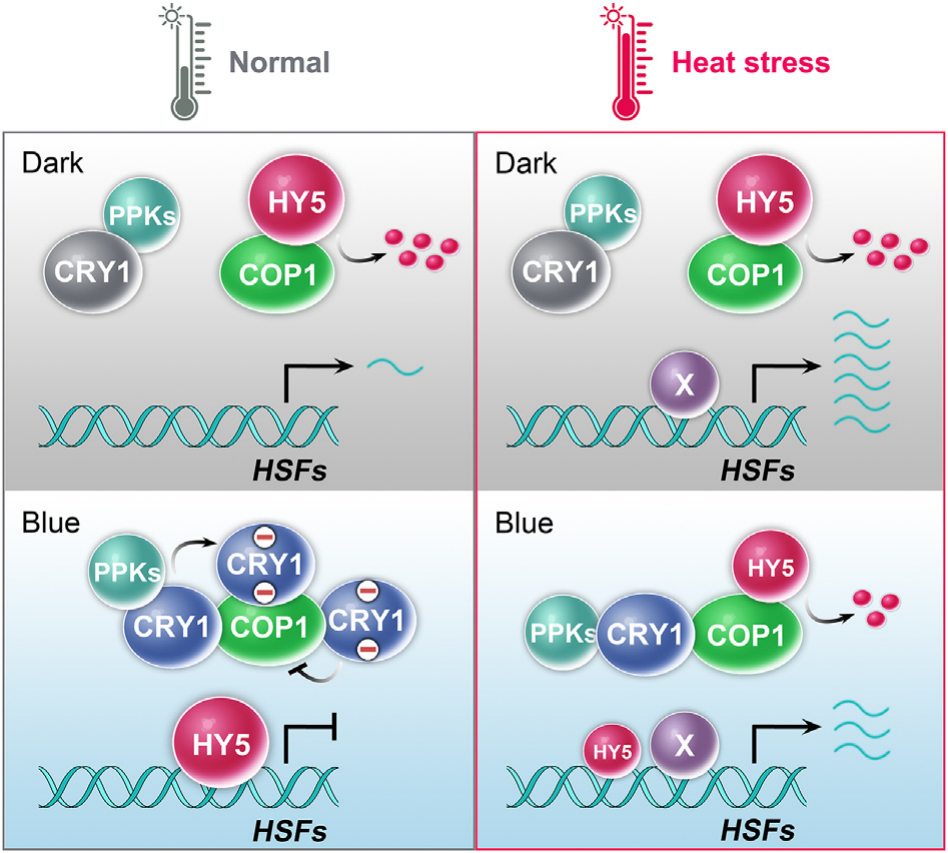
A working model of the CRY1–COP1–HY5 axis regulating plant heat-stress responses. In the dark, COP1 mediates degradation of HY5 protein under both normal and high temperatures. *HSFs* are highly expressed in response to heat stress in the dark. In the presence of blue light at normal temperatures, CRY1 undergoes blue-light-dependent phosphorylation by PPKs. This phosphorylation enhances the CRY1–COP1 interaction, resulting in suppression of COP1 activity and accumulation of HY5. The accumulated HY5 proteins bind to the promoters of *HSFs*, suppressing their transcription under normal temperatures in blue light. When the temperature rises to stressful levels, heat stress inactivates PPKs. As a result, CRY1 phosphorylation is hindered, leading to weakened suppression of COP1 activity. This leads to excessive degradation of HY5 and increased expression of *HSFs* compared with that in blue light at normal temperatures.

In this study, we investigated how blue light and the photoreceptor CRY1 affect heat-shock response and thermotolerance in *Arabidopsis*. On the basis of the experimental results, we hypothesize that CRY1 mediates blue-light suppression of thermotolerance by the CRY1–COP1–HY5 module (Figure 7). According to this hypothesis, COP1 mediates degradation of the HY5 protein in darkness at both normal and high temperatures. In darkness, *HSFs* are highly expressed in response to heat stress. In blue light at normal temperature, CRY1 undergoes blue-light-dependent phosphorylation by PPKs to enhance the CRY1–COP1 interaction, resulting in suppression of COP1 activity, accumulation of HY5, and low expression levels of *HSFs*. However, plants exposed to blue light escape HY5 suppression of *HSF* expression in response to stressful heat-shock temperatures, resulting in increased expression of *HSFs* and thermotolerance. Our results show that the heat inactivation of PPKs and CRY1 phosphorylation can explain, at least in part, the blue-light- and heat-dependent induction of *HSF* expression and thermotolerance. This is because unphosphorylated CRY1 exhibits weaker interaction with COP1, resulting in excessive degradation of HY5 and increased expression of *HSFs* compared with that under blue light at normal temperatures. Our hypothesis appears to reveal an important function of CRY1 as a “safety valve” that can prevent excessive *HSF*expression and excessive stress responses when plants are exposed to light that is usually accompanied by increased but not lethally high temperatures. On the other hand, inhibition of CRY1 activity at lethally high temperatures can “turn off” this safety valve to allow expression of *HSFs* high enough to ensure plant survival at lethally high temperatures. Consistent with the previous discovery of a similar safety valve function of the CRY2–COP1–HY5 module under cold temperatures (Li et al., 2021), our study provides further evidence for the crucial roles of the CRY1–COP1–HY5 module in the light regulation of plant thermotolerance. Importantly, CRY1 not only inhibits the expression of positive regulators of thermotolerance, such as *HSFA1D*, but also suppresses the expression of negative regulators of thermotolerance, such as *HSFB1* (Figure 2C). Therefore, CRY1 may orchestrate the balance between positive and negative regulators of thermotolerance to fine-tune the overall heat-stress response.

In addition to the CRY1–COP1–HY5 module, CRY1 may also regulate plant thermotolerance by other mechanisms. For example, CRY1 may directly interact with HSFs to alter thermotolerance via a mechanism distinct from that reported here (Gao et al., 2023). We noticed that only 12% of the CRY1-regulated genes were shared between normal and heat-stress temperatures under our experimental light conditions, which is consistent with the existence of additional mechanism(s) underlying CRY1-mediated light regulation of thermotolerance.

In the present study, we focused on the effect of CRY1-mediated suppression of thermotolerance. However, our results do not exclude the possibility that CRY2 may participate in the blue-light regulation of thermotolerance under different conditions. Moreover, other photoreceptors, especially phytochromes, are also known to participate in the light regulation of plant thermotolerance (Song et al., 2017; Arico et al., 2019). For example, the red-light receptor phyB is known to act as a temperature sensor, with ambient warm temperatures promoting its inactivation (Jung et al., 2016; Legris et al., 2016). Whether CRYs may act as temperature sensors in the heat-shock response is not clear. In fact, most thermal sensing studies of plant CRYs have been performed under ambient warm temperatures, and thermal sensing under heat stress is not well understood at present. Exploring whether photoreceptors function as thermal sensors in response to heat stress would be an interesting area of future research.

Plant photoresponses and thermotolerance have each been studied extensively, with many aspects of their molecular mechanisms clarified. However, understanding how light response affects thermotolerance in plants poses a more challenging problem that has not been well studied or understood. Given the almost countless light and temperature combinations plants may encounter in nature, researchers have examined the effects of light and photoreceptors on thermotolerance under various conditions, such as different light or photoperiod pretreatments, different acclimation conditions, and different heat-treatment regimens (Song et al., 2017; Arico et al., 2019; Han et al., 2019; Gao et al., 2023). Although these diverse experimental conditions are necessary to explore real-life plant responses, they also make it difficult to directly compare the results from different studies. For example, similar to our findings in this study, light inhibition of thermotolerance has also been reported by others under different experimental conditions (Song et al., 2017; Arico et al., 2019). However, the effects of light or photoreceptors on thermotolerance may vary significantly or even show opposite results under other conditions. For example, in contrast to our results, CRY1 was recently reported to promote thermotolerance (Gao et al., 2023). We do not yet have a satisfactory explanation for these discrepancies, although it is conceivable that they may result from differences in experimental systems. For example, there were some notable differences in experimental conditions between the two studies, including differences in starting materials (etiolated seedlings vs. continuous-light-grown seedlings), pretreatment conditions (1 h vs. 24 h blue light), length of heat treatment (2 h vs. 3 h or longer), and recovery conditions (long-day photoperiod vs. continuous light). These different experimental conditions and results appear to reinforce our intuition that photomorphogenesis, the duration of light or heat exposure, the circadian clock, and photoperiod all significantly affect thermotolerance. This underscores the necessity for additional studies to better understand how light regulates thermotolerance in plants.

## Methods

### Plant materials and growth conditions

The *GFP-CRY1/cry1cry2rdr6*, *NLS-GFP-CRY1/cry1cry2rdr6*, *CRY1-7A/cry1cry2rdr6*, *CRY1-10A/cry1cry2rdr6*, *CRY1-7D/cry1cry2rdr6*, *CRY1-10D/cry1cry2rdr6*, *GFP-CRY1/4myc-CRY1*, and *4myc-CRY1/cry1cry2rdr6* transgenic lines have been described previously (Gao et al., 2022; Liu et al., 2022). To prepare transgenic overexpression lines, *proHY5::HY5-Flag-GFP* and *pACT2::HY5-Flag-GFP* were introduced into *rdr6-11* (Peragine et al., 2004).

For routine maintenance, *Arabidopsis* was grown under long-day conditions (16 h light/8 h dark) at 22°C. All seedlings were grown on Murashige and Skoog (MS) medium with 1% sucrose.

### Plasmid construction

All plasmid constructs in this study were generated using the In-Fusion Cloning method. *pFGFP* binary vectors, as described previously (Liu et al., 2022), were used for the creation of transgenic overexpression lines. The coding sequence regions of *HY5* and the 756-bp *HY5* promoter (*proHY5*), including the 5′ untranslated region, were seamlessly connected and cloned into the SacI/SpeI-digested *pFGFP* vector to generate the *proHY5::HY5-Flag-GFP* construct. The *HY5* coding sequence was cloned into the SpeI-digested *pFGFP* vector to generate the *pACT2::HY5-Flag-GFP* construct. Split-LUC constructs were created using nLUC and cLUC vectors as described previously (Chen et al., 2021). The coding sequences of *COP1* or *HY5* were fused to nLUC, and the coding sequences of *COP1* or *CRY1* were fused to cLUC. The BiFC constructs were created using the dual-transgenic BiFC vector *pDTQ27*, which is derived from the previously published *pDT1* vector (He et al., 2016). This vector enables the simultaneous expression of two BiFC proteins within a single vector. In these constructs, PPK1 or COP1 were fused with the N-terminal half of yellow fluorescent protein (nYFP), and CRY1 was fused with the C-terminal half of yellow fluorescent protein (cYFP). In the dual-LUC assay, the following promoter regions were fused to the firefly LUC gene in the *pGreenII 0800-LUC* plasmid (Hellens et al., 2005): a 1.5-kb promoter region from *HSFA2*, a 1-kb promoter region from *HSFA7A*, or a 1-kb promoter region from *HSFA7B*.

### Thermotolerance assay

In our study, the initial growth condition of the seedlings is referred to as the basal growth condition. After growing in the basal growth condition for 4–5 days at 22°C, seedlings were pretreated under different light conditions for 1 day at 22°C. Subsequently, the seedlings were subjected to heat-shock treatment at 44°C for the indicated times under the same light conditions as the pretreatment. After the heat shock, the seedlings were allowed to recover at 22°C for 5 days under continuous-light conditions. The survival rate, defined as the percentage of healthy seedlings, was calculated at the end of the recovery period. Surviving seedlings were defined as those that continued to grow normally (greenish) after heat shock, whereas dead seedlings were those that failed to grow normally (yellowish or whitish) after heat shock. The intensities of light used in the thermotolerance assays were as follows: blue light, 100 μmol m^−2^ s^−1^ or 1 μmol m^−2^ s^−1^; red light, 100 μmol m^−2^ s^−1^; and white light, 100 μmol m^−2^ s^−1^. Seedlings of different genotypes were assayed for thermotolerance on both the same Petri dish and different Petri dishes to avoid experimental bias. At least three independent biological repeats were performed for each thermotolerance assay shown in the main figures.

### RNA sequencing and data processing

Samples were prepared for RNA sequencing from seedlings pre-grown at 22°C in continuous white light (100 μmol m^−2^ s^−1^) for 4 days and then pretreated with blue light for 1 day, followed by a heat-stress treatment at 44°C for 2 h (referred to as 44°C) or without a heat treatment (referred to as 22°C). Total RNA samples from three independent biological replicates were subjected to rRNA-depleted RNA sequencing at Berry Genomics (http://www.berrygenomics.com/). The sequencing reads were adaptor trimmed and quality filtered with Trimmomatic (v.0.39) (Bolger et al., 2014), followed by alignment and quantification using RSEM (v.1.3.1) (Li and Dewey, 2011) with default parameters. The *Arabidopsis* genome sequence from TAIR (version 10) served as the reference genome. Values of fragments per kilobase of transcripts per million mapped reads (FPKM) were obtained through the RSEM analysis. Genes with a log_2_(FPKM) value greater than −5 were considered to be expressed genes. Statistical analysis of differentially expressed genes was performed with a *p*-value threshold of <0.05 and an FPKM fold-change cutoff of ±2. GO enrichment analysis was performed in RStudio using the enrichplot R package. GSEA was performed using the R packages cluster Profiler, enrichplot, org.At.tair.db, and DOSE (Subramanian et al., 2005; Reimand et al., 2019; Wu et al., 2021). Gene sets identified by GSEA were required to meet specific criteria, including an absolute value of normalized enrichment score greater than 1 and an adjusted *p*-value less than 0.05. For data visualization, Venn diagrams and heatmaps were constructed using TBtools (Chen et al., 2020), and additional data visualization was performed using R-Studio.

### Transient expression in tobacco

The transient expression assays in tobacco were performed following a previously described protocol with some modifications (Zhang et al., 2020). For tobacco infiltration, each assay involved at least three independent plants, with two or three leaves infiltrated per plant. The infiltrated tobacco was subjected to a temperature treatment, being incubated at 44°C for 1 h for further analysis.

For split-LUC assays, 1 mM D-luciferin with 0.01% TritonX-100 was sprayed onto the leaves prior to analysis. The leaves were then kept in the dark for 10 min before signal detection using a Tanon 5200 instrument. Luminescence intensity was quantified using ImageJ. For dual-LUC assays, luminescence was quantified using the Dual-Luciferase Reporter Assay System (cat. no. E1960, Promega).

### Co-IP and Pi assay in HEK293T cells

The HEK293T cell experiments were performed following established procedures with some minor adjustments (Chen et al., 2021). Plasmid DNA (5 μg) was mixed with 15 μl of Polyethylenimine Linear MW40000 (1 mg/ml) (cat. no. 40816ES03, Yeasen) in 300 μl of Opti-MEM medium. The mixture was vortexed and incubated at room temperature for 15 min before being applied to the cells. Cells were typically harvested 36–48 h after transfection. The co-IP experiments using HEK293T samples were performed in accordance with previously described methods (Chen et al., 2021).

### EMSAs

EMSAs were performed using Cy5-labeled probes as described previously, with minor modifications (Yang et al., 2023). The sequences of the DNA probes were as follows: *HSFA2 G-box*, GTCTTGTCGCCACGTGCTCACATAAA; *HSFA2 HSE*, GATTAGTAACGAAGTTTCTGGAACATTGTCTTGTCTTGTCGCCACGTGCTCACATAAA; and *HSFA7A G-box*, TTACCTTTTGTGACGTGTTAACTTA. Purified His-HY5 proteins were incubated with DNA probes at 25°C or 44°C for 30 min. Signals were assayed with an Odyssey CLx infrared imaging system (Li-Cor Biosciences).

### ChIP assays

ChIP assays were performed as described previously (Yang et al., 2023). About 2 g of *proHY5::HY5-GFP* and control *pACT2::GFP* seedlings grown in blue light (100 μmol m^−2^ s^−1^) at 22°C for 10 days were harvested for each ChIP–qPCR analysis using GFP-trap beads.

### Protein extraction and immunoblotting

Protein extraction, immunoblotting, and intensity quantification were performed as described previously (Chen et al., 2021). The primary and secondary antibodies used in this study were as reported previously (Liu et al., 2022), except for anti-Actin (1:2000, code M20009M, Abmart), anti-HY5 (1:1000, gift from Dr. Jigang Li; cat. no. PHY1908, PHYTOAB), and anti-PPK1 (1:800, homemade).

### Accession numbers

The accession numbers of genes mentioned in this study are as follows: *CRY1*, AT4G08920; *COP1*, AT2G32950; *HY5*, AT5G11260; *PPK1*, AT1G77720; *PPK3*, AT3903940; *PPK4*, AT2G25760; *HSFA2*, AT2G26150; *HSFA7A*, AT3G51910; and *HSFA7B*, AT3G63350.

The mutant plants used in this study were as follows: *cry1-304* (Mockler et al., 1999), *cop1-4* (McNellis et al., 1994), *cop1-6* (McNellis et al., 1994), *hy5-215* (Oyama et al., 1997), *hsp101* (Tong et al., 2022), *cop1-4hy5-215* (gift from Dr. Jigang Li), and *cry1cop1-4* (genetic cross performed in this study).

### Statistical analysis

One-way ANOVA followed by Tukey’s multiple comparison test or two-way ANOVA followed by Sidak’s multiple comparison test were used for statistical analysis in GraphPad Prism 8 software.

## Data availability

The data and materials from this study are available from the corresponding author upon reasonable request. Sequencing data are available at the National Center for Biotechnology Information (NCBI) Gene Expression Omnibus (GEO) database (GEO: GSE254813).

## Funding

This work is supported by the National Key Research and Development Program of China (2020YFA0509700), the Natural Science Foundation of Fujian Province (2024J011015 and 2023J01485), the National Natural Science Foundation of China (32472070), and the Natural Science Foundation of Guangdong Province (2022A1515011002).

## Acknowledgments

We thank Dr. Chentao Lin for discussions, Dr. Jigang Li for providing the endogenous HY5 antibody and *cop1-4hy5-215* double mutants, and Dr. Weiqiang Qian for sharing the *hsp101* mutants. No conflict of interest is declared.

## Author contributions

S.L. and Qin Wang conceived and designed the experiments; S.L., Qiongli Wang, M.Z., Y.W., and M.Y. performed the experiments; J.Z. and G.L. performed bioinformatics analysis; S.L. and Qin Wang wrote the paper; J.Z. and Qiongli Wang reviewed and edited the paper.

## Supplemental information

Document S2. Dataset S1–S8

Document S3. Article plus supplemental information
