# Supplementary material for: The CRY1–COP1–HY5 axis mediates blue-light regulation of Arabidopsis thermotolerance
Source: Plant Commun. 2025 Jan 29;6(4):101264. doi: 10.1016/j.xplc.2025.101264 (PMC12010382; doi:10.1016/j.xplc.2025.101264)
Supplement: Document S1. Figures S1–S15 [file mmc1.pdf]

Plant Communications, Volume *6*

Supplemental information

The CRY1–COP1–HY5 axis mediates blue-light regulation of *Arabidopsis* thermotolerance

Siyuan Liu, Qiongli Wang, Ming Zhong, Guifang Lin, Meiling Ye, Youren Wang, Jing Zhang, and Qin Wang

Supplemental Information

Article title: **The CRY1-COP1-HY5 axis mediates blue-light regulation of Arabidopsis thermotolerance**

Authors: Siyuan Liu, Qiongli Wang, Ming Zhong, Guifang Lin, Meiling Ye, Youren Wang, Jing Zhang, Qin Wang

Supplemental Figure 1. The blue-light fluence-dependent inhibition of Arabidopsis thermotolerance.

Supplemental Figure 2. CRY1 mediates blue-light suppression of Arabidopsis thermotolerance independently of the basal growth light conditions and seedling architecture.

Supplemental Figure 3. CRY1 mediates blue-light suppression of Arabidopsis thermotolerance.

Supplemental Figure 4. Summary of RNA-seq reads across samples.

Supplemental Figure 5. CRY1 regulates both light-signaling pathways and heat stress response pathways.

Supplemental Figure 6. Heat stress does not affect the photoactivation of CRY1.

Supplemental Figure 7. Heat stress inhibits the phosphorylation of CRY1.

Supplemental Figure 8. Heat stress inhibits the blue light-dependent degradation of CRY1.

Supplemental Figure 9. The thermotolerance phenotypes of CRY1 phospho-mutants in continuous blue light and red light.

Supplemental Figure 10. Confocal images displaying the reduced HY5 GFP fluorescence in response to heat stress in cotyledon.

Supplemental Figure 11. COP1 is responsible for HY5 ubiquitination under heat stress.

Supplemental Figure 12. The thermotolerance phenotypes of *cop1* and *hy5* mutants under continuous light conditions.

Supplemental Figure 13. Snapshots displaying the binding of HY5 to the promoters of *HSF* genes.

Supplemental Figure 14. The expression of HY5-GFP in tobacco transient experiments in Figure 6.

Supplemental Figure 15. HY5 does not regulate the expression of *HSFB3* and *HSFB4*.

The following Datasets are available in a separate excel file:

Supplemental Dataset 1. Heat-upregulated genes in WT (WT-44 vs WT-22).

Supplemental Dataset 2. The expression of *HSF* and *HSP* genes in WT and *cry1* at 22°C and 44°C.

Supplemental Dataset 3. GO enrichment analysis of the heat-upregulated genes in WT.

Supplemental Dataset 4. GSEA analysis of gene sets in response to heat in WT.

Supplemental Dataset 5. CRY1-regulated genes at 22°C (*cry1*-22 vs WT-22).

Supplemental Dataset 6. CRY1-regulated genes at 44°C (*cry1*-44 vs WT-44).

Supplemental Dataset 7. CRY1-regulated biological processes at 22°C (*cry1*-22 vs WT-22).

Supplemental Dataset 8. CRY1-regulated biological processes at 44°C (*cry1*-44 vs WT-44).

Supplemental Figure 1

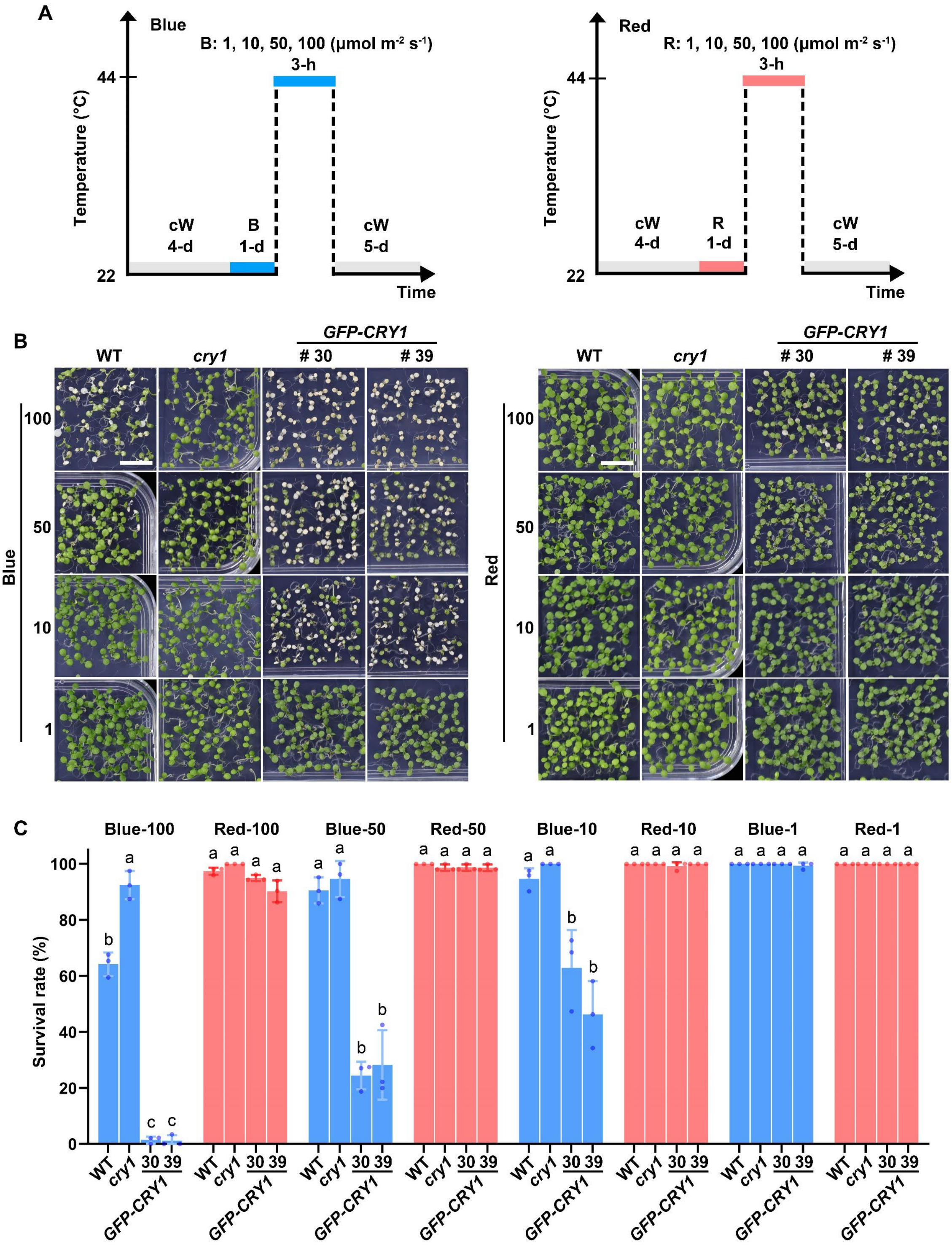

Supplemental Figure 1. The blue-light fluence-dependent inhibition of Arabidopsis thermotolerance.

(A) Representative charts showing heat stress experimental conditions. Plants were initially grown on MS plates at 22°C in continuous white light (cW, 100 µmol m^{-2} s^{-1}) for 4 days. Subsequently, they were pretreated with different fluences of blue light (B, 1, 10, 50, 100 µmol m^{-2} s^{-1}), red light (R, 1, 10, 50, 100 µmol m^{-2} s^{-1}) for 1 day at 22°C before subjecting to heat stress treatment at 44°C for 3 hours. Following the heat stress treatment, seedlings were allowed to recover for 5 days at 22°C under continuous white light (100 µmol m^{-2} s^{-1}).

(B) Representative thermotolerance phenotypes of the indicated genotypes are displayed. Scale bar, 1 cm.

(C) Quantification of the survival rates of seedlings in (B). Different letters indicate statistically significant differences of survival rates between genotypes within each treatment (one-way ANOVA followed by Tukey's multiple comparisons test, $p < 0.05$).

Supplemental Figure 2

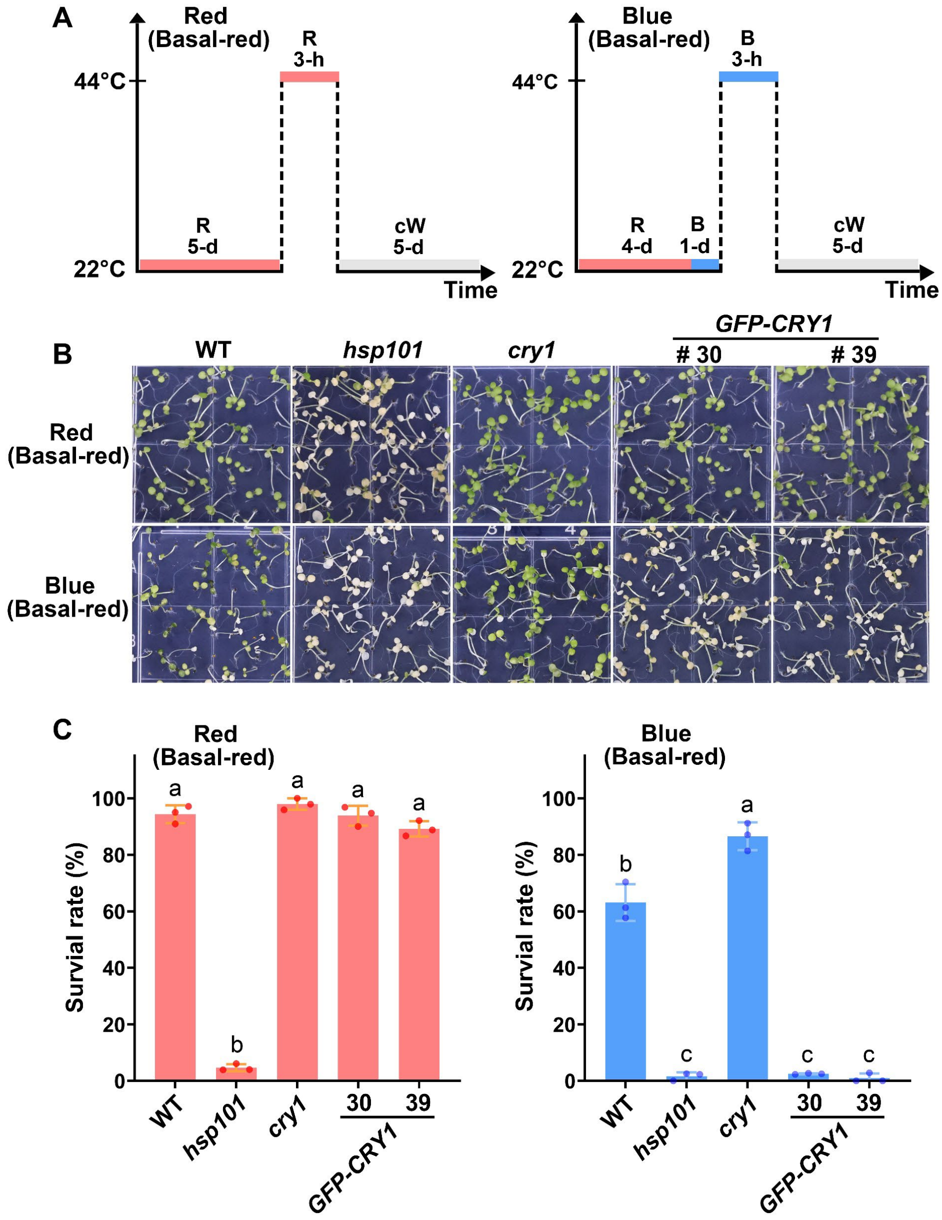

Supplemental Figure 2. CRY1 mediates blue-light suppression of Arabidopsis thermotolerance independently of the basal growth light conditions and seedling architecture.

(A) Representative charts showing heat stress treatment conditions. Plants were initially grown on MS plates at 22°C in red light (Basal-red, 100 μmol m^{-2} s^{-1}) for 4 days. Subsequently, they were kept in red light or pretreated in blue light (B, 100 μmol m^{-2} s^{-1}) for 1 day before subjecting to heat stress treatment at 44°C for 3 hours. Following the heat stress treatment, seedlings were allowed to recover for 5 days at 22°C under continuous white light.

(B) Representative thermotolerance phenotypes of the indicated genotypes are displayed.

(C) Quantification of the survival rates of seedlings in (B). The data are presented as the mean ± SD of three biological replicates, with approximately 50 plants per genotype examined in each biological replicate. Different letters indicate statistically significant differences of survival rates between genotypes (one-way ANOVA followed by Tukey's multiple comparisons test, $p < 0.05$).

Supplemental Figure 3

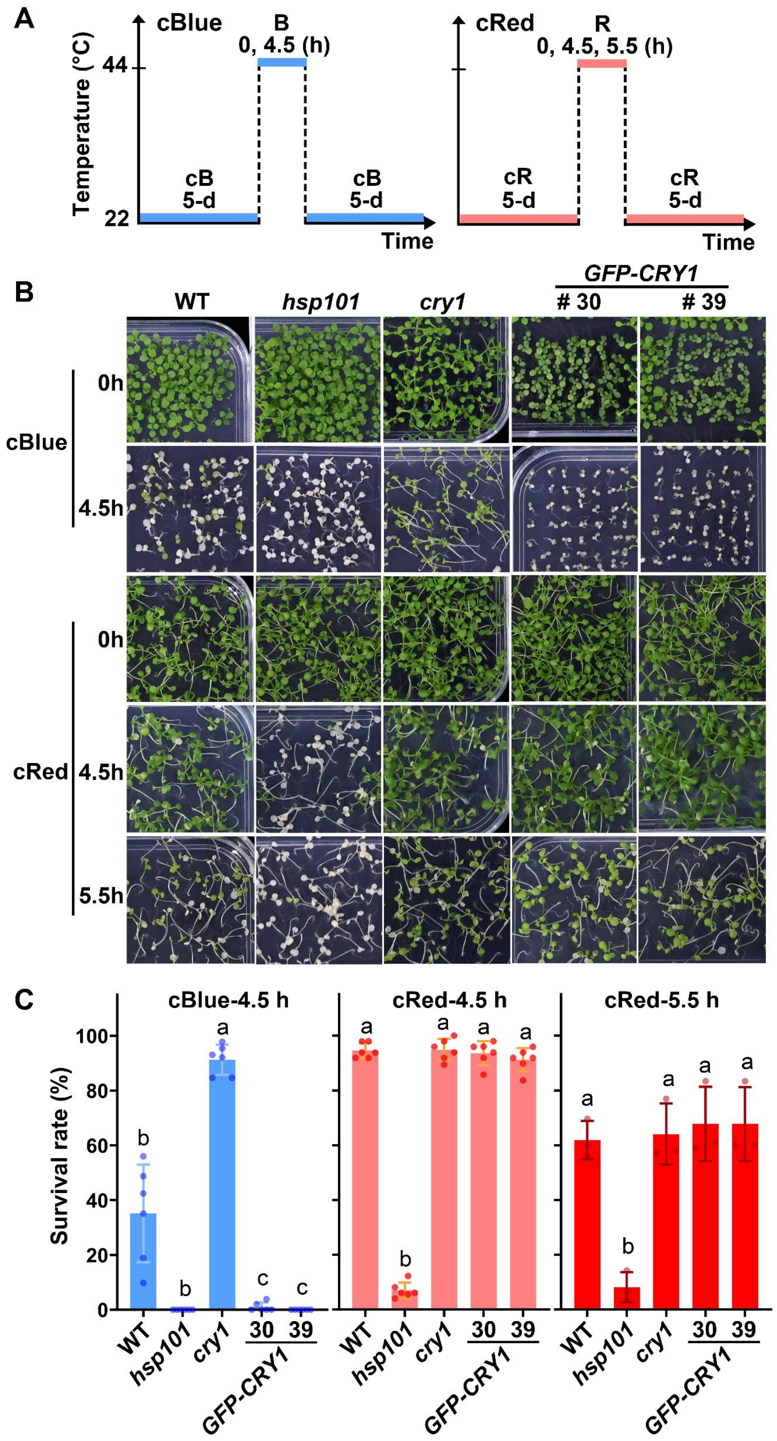

Supplemental Figure 3. CRY1 mediates blue-light suppression of Arabidopsis thermotolerance.

(A) Representative charts showing heat stress treatment conditions. Plants were initially grown on MS plates at 22°C under continuous blue light (cB, 100 μmol m^{-2} s^{-1}) or continuous red light (cR, 100 μmol m^{-2} s^{-1}) for 5 days. Subsequently, they were subjected to a heat stress treatment at 44°C for 0, 4.5, or 5.5 hours at day-5, followed by a recovery period of 5 days at 22°C under respective growth light conditions.

(B) Representative thermotolerance phenotypes of the indicated genotypes are displayed.

(C) Quantification of the survival rates of seedlings in (B). The data are presented as the mean ± SD of at least three biological replicates, with approximately 50 plants per genotype examined in each biological replicate. Different letters indicate statistically significant differences of survival rates between genotypes within each treatment (one-way ANOVA followed by Tukey's multiple comparisons test, $p < 0.05$).

Supplemental Figure 4

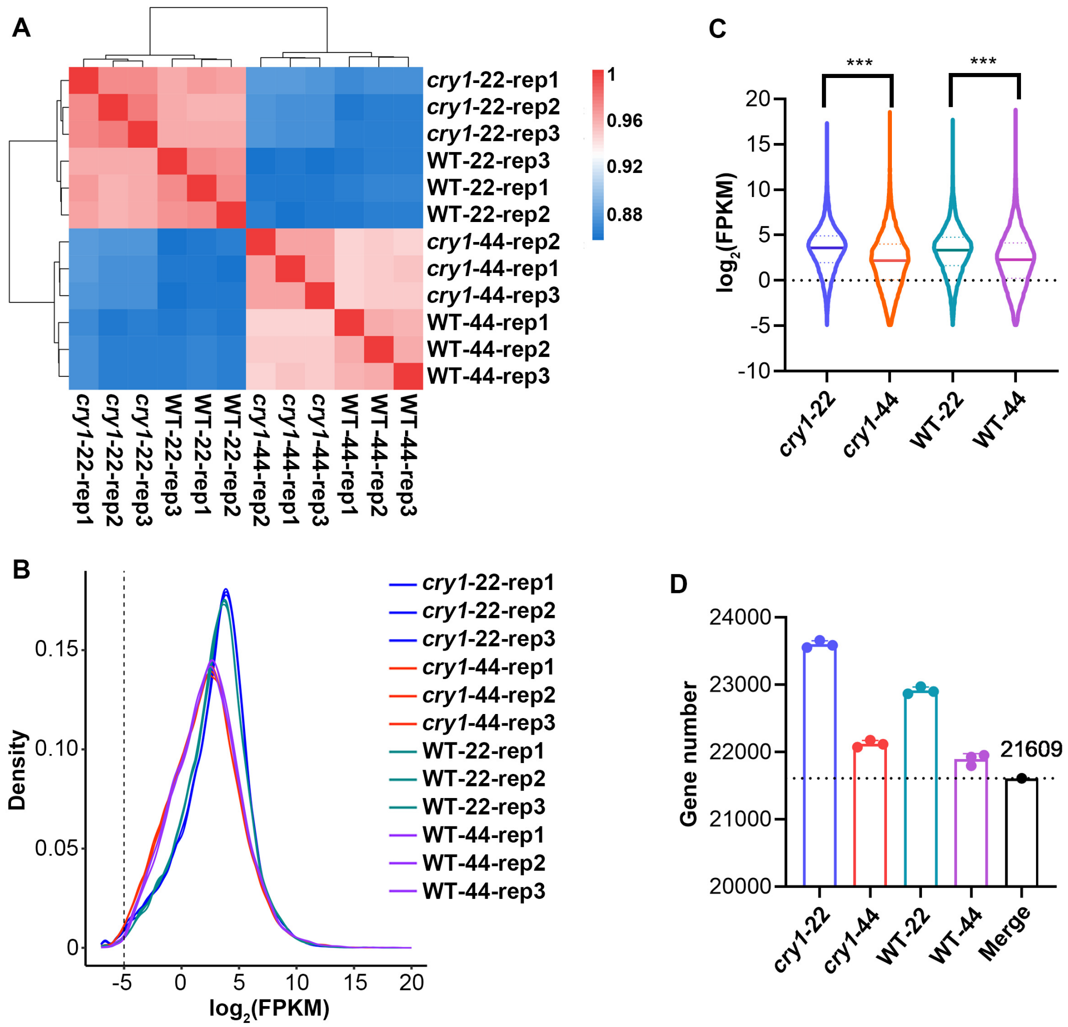

Supplemental Figure 4. Summary of RNA-seq reads across samples.

(A) Pearson correlation analysis of gene expression between different conditions in WT or *cry1* mutant. Plants were grown on MS plates at 22°C in continuous white light (100 μmol m^{-2} s^{-1}) for 4 days, then acclimated in blue light (100 μmol m^{-2} s^{-1}) for 1 day. RNA-seq samples were collected after heat stress treatment at 44°C for 2 hours under blue light (referred to as 44°C) or collected at 22°C under blue light (referred to as 22°C).

(B) Overview of the FPKM density in WT and *cry1* under 22°C and 44°C. Genes with log2 (FPKM) greater than -5 are considered expressed genes.

(C) Distribution of FPKM values across samples. ****p* < 0.001, Student's t-test.

(D) Number of expressed genes across samples. The means and standard deviates are shown (n = 3). The merged data represent genes expressed across all four samples, which were used for subsequent bioinformatics analysis.

Supplemental Figure 5

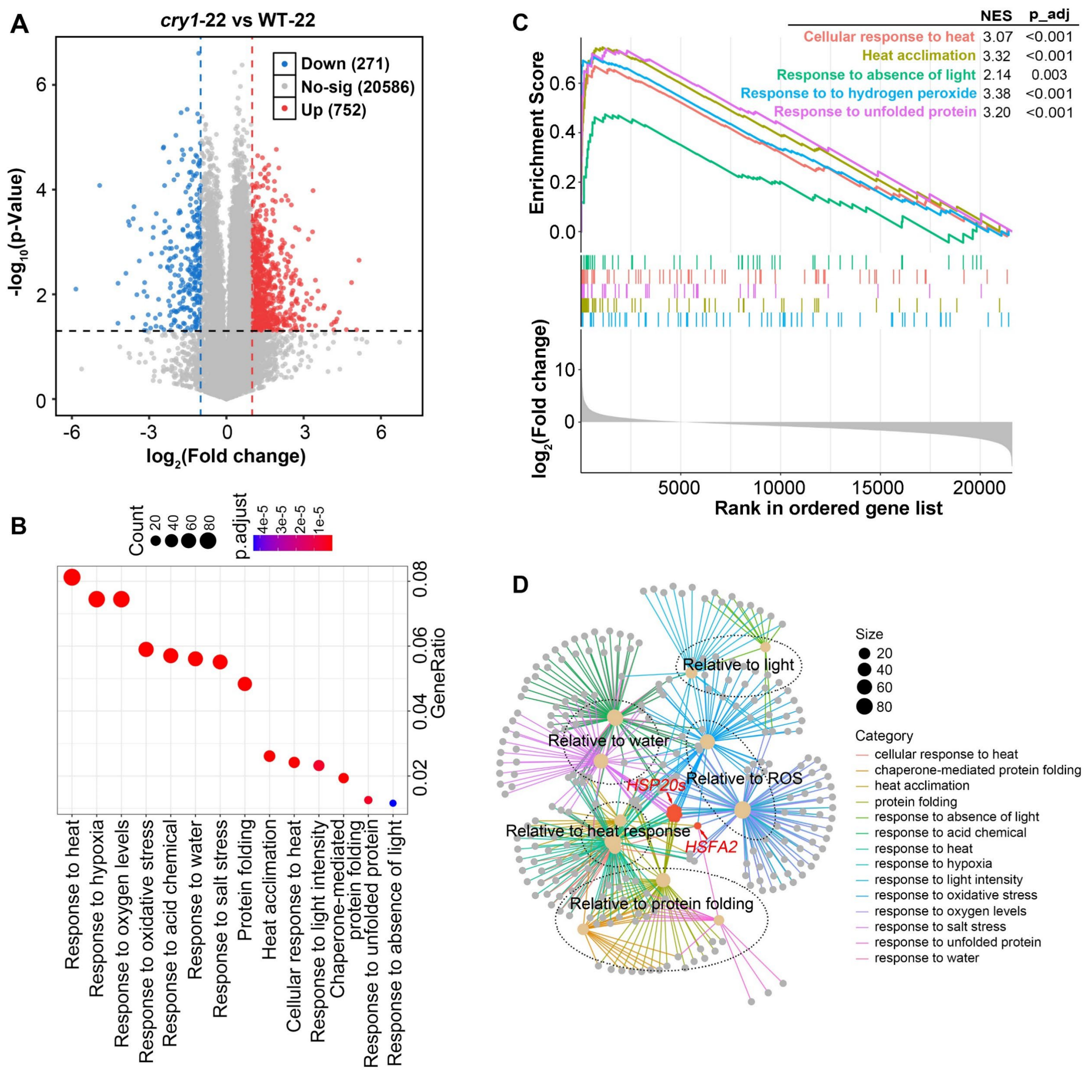

Supplemental Figure 5. CRY1 regulates both light signaling pathways and heat stress response pathways.

(A) CRY1-regulated genes at 22°C. Differential expressed genes were defined as $|\log_2(\text{fold change})| \geq 1$, $p < 0.05$). Down, downregulated genes; No-sig, no significant changed genes; Up, upregulated genes.

(B) GO enrichment analysis of the upregulated genes in wild-type in response to heat. The top 15 biological processes are shown.

(C) Gene set enrichment analysis of genes in response to heat in WT. Significantly enriched heat-, light- and stress-responsive gene sets are shown (NES>1, adjusted $P < 0.05$). NES, normalized enrichment score.

(D) Weighted interaction network of heat upregulated genes enriched in (C). The central heat-induced genes, *HSFA2* and *HSP20s*, are highlighted in red.

Supplemental Figure 6

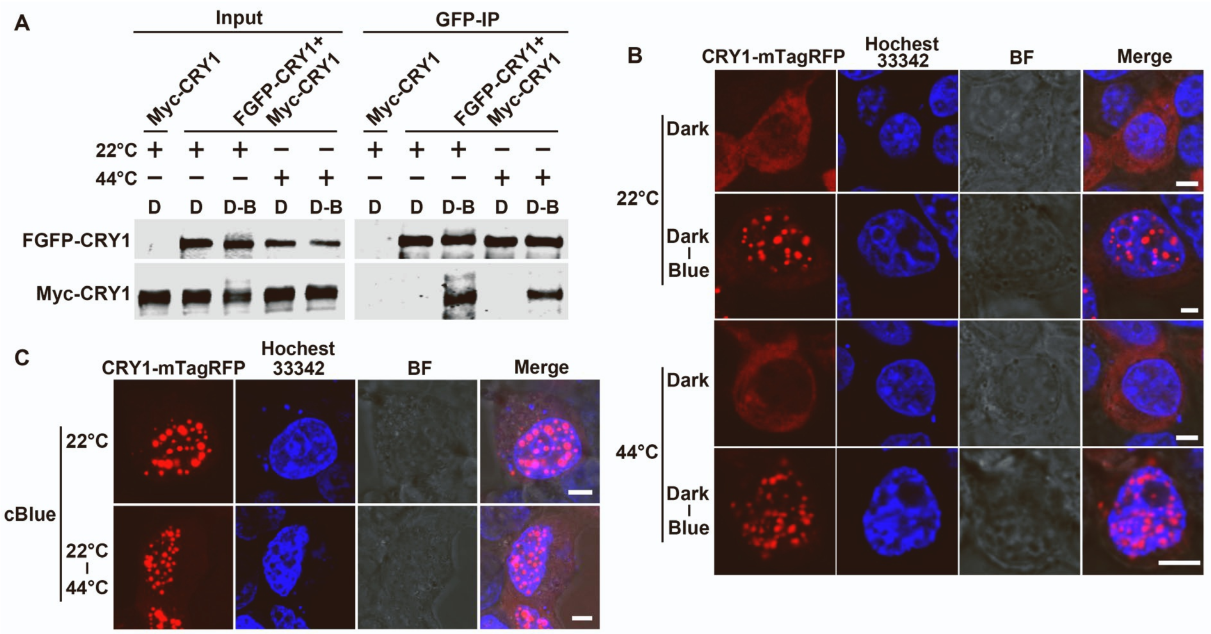

Supplemental Figure 6. Heat stress does not affect the photoactivation of CRY1.
(A) Co-IP results showing the photo-oligomerization of CRY1 at 22°C and 44°C. 10-day-old etiolated seedlings co-expressing *FGFP-CRY1* and *Myc-CRY1* were maintained in darkness (D) or exposed to 100 μmol m^{-2} s^{-1} of blue light for 1 hour (D-B) at 22°C and 44°C before sample collection. GFP-Trap beads were used for immunoprecipitation, and FGFP-CRY1 and Myc-CRY1 were detected using anti-Flag and anti-Myc antibodies, respectively.
(B,C) Confocal images illustrating the formation of CRY1 nuclear photobodies in response to heat stress. HEK293T cells expressing CRY1-mTagRFP were kept in darkness or exposed to blue light (100 μmol m^{-2} s^{-1}) for 1 hours at 22°C and 44°C before fixation in 4% paraformaldehyde before imaging (B). HEK293T cells expressing CRY1-mTagRFP were kept in continuous blue light (100 μmol m^{-2} s^{-1}) for 1 day at 22°C, and then treated under 44°C for 1 hour (22°C-44°C) before fixation in 4% paraformaldehyde before imaging (C). Hoechst 33342 staining was used to indicate the nuclei. BF, bright field; scale bar, 5 μm.

Supplemental Figure 7

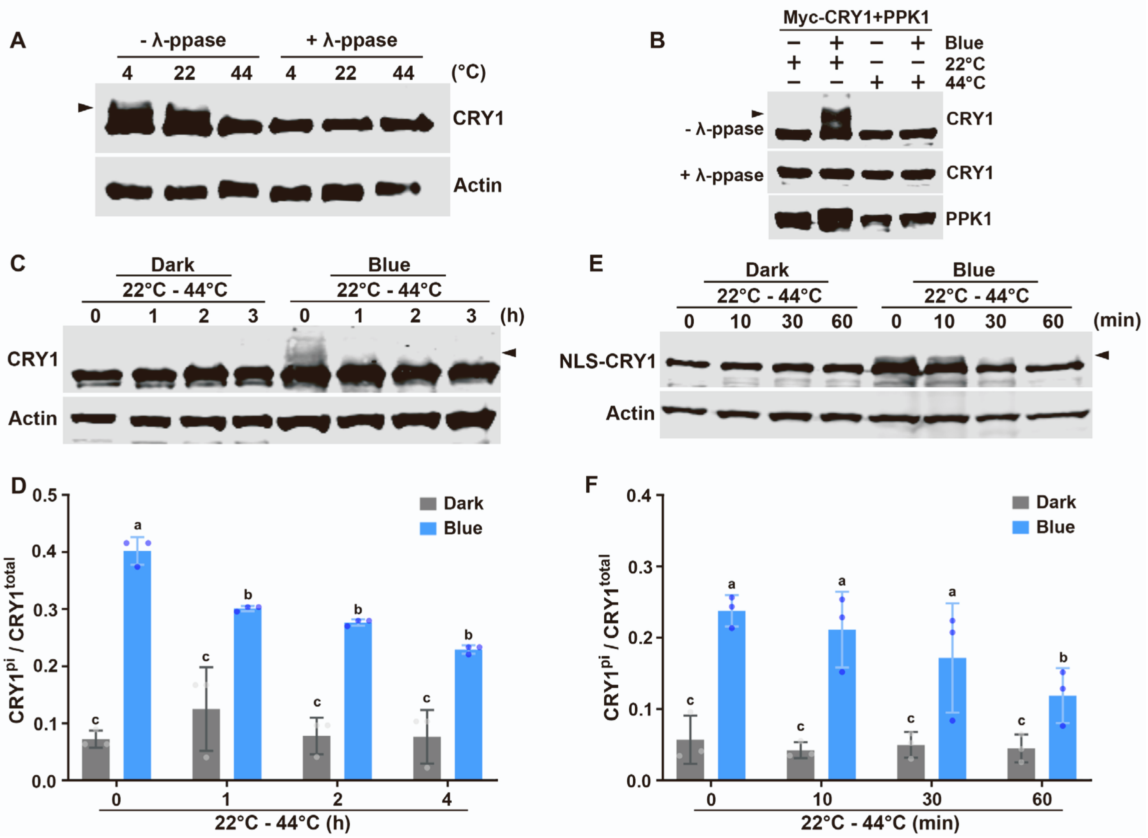

Supplemental Figure 7. Heat stress inhibits the phosphorylation of CRY1.

(A) 5-day-old wild-type seedlings grown at 22°C under constant blue light (100 μmol m^{-2} s^{-1}) were treated at 4°C, 22°C, 44°C for 4 hours. Plant lysates were treated with λ-PPase (+ λ-PPase) or without (- λ-PPase).

(B) HEK293T cells co-expressing Flag-PPK1 with Myc-CRY1 were kept in the dark (- Blue) or exposed to blue light (100 μmol m^{-2} s^{-1}, + Blue) for 1 hour at 22°C or 44°C. The levels of PPK1 and CRY1 were detected using anti-PPK1 and anti-CRY1 antibodies, respectively.

(C, E) 5-day-old wild-type (C) or *NLS-GFP-CRY1* overexpression (E) seedlings, grown under constant darkness or blue light (100 μmol m^{-2} s^{-1}), were transferred to 44°C for the indicated time under the same light conditions. The levels of CRY1 and Actin were detected using anti-CRY1 and anti-Actin 2 antibodies, respectively, with Actin serving as the loading control.

(D, F) Quantification of CRY1 phosphorylation in response to heat stress in (C) or (E), respectively. The degree of CRY1 phosphorylation was determined by normalizing phosphorylated CRY1 to total CRY1 ($CRY1^{pi}/CRY1^{total}$) and is presented as the mean ± SD (n=3 individual immunoblots). Different letters indicate statistically significant differences between samples under darkness and blue light (two-way ANOVA followed by Sidak's multiple comparisons test, $p < 0.05$).

Arrowheads indicate phosphorylated CRY1.

Supplemental Figure 8

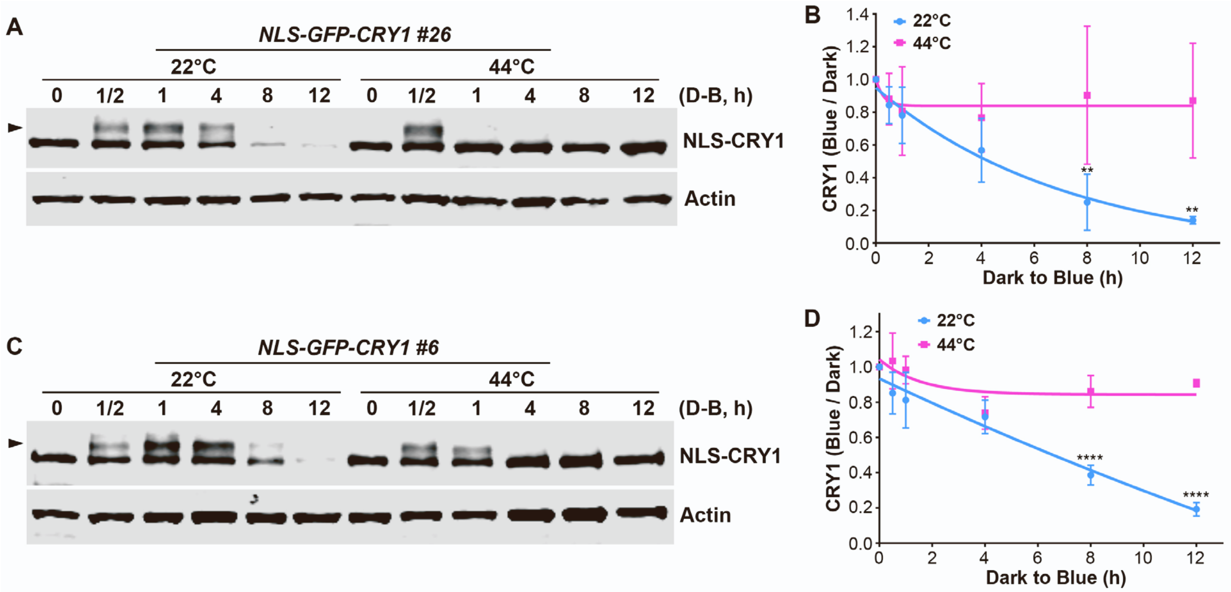

Supplemental Figure 8. Heat stress inhibits the blue light-dependent degradation of CRY1.

(A, C) 7-day-old etiolated seedlings irradiated with 100 μmol m^{-2} s^{-1} of blue light under 22°C or 44°C for the indicated time. Anti-CRY1 antibody and anti-Actin antibody were used to detect CRY1 and Actin, respectively.

(B, D) Quantitative analysis of NLS-GFP-CRY1 degradation in (A) or (C), respectively. CRY1 (B/D) = $(CRY1/Actin)^{blue}$ / $(CRY1/Actin)^{dark}$. Data are presented as mean ± SD (n=3 individual immunoblots). The best-fitted curves with one-phase decay of nonlinear regression are shown.

Supplemental Figure 9

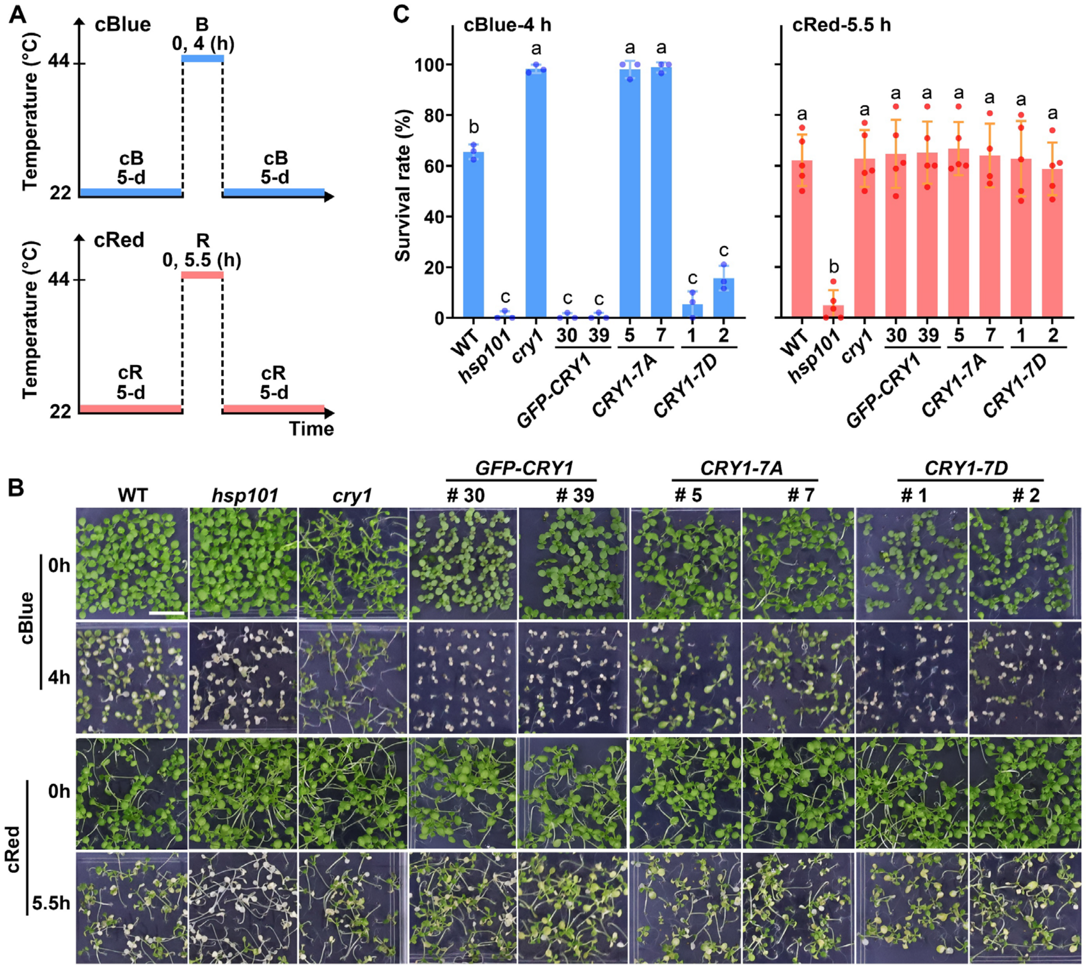

Supplemental Figure 9. The thermotolerance phenotypes of CRY1 phospho-mutants in continuous blue light and red light.

(A) Representative charts showing heat stress treatment conditions. Plants were initially grown on MS plates at 22°C under continuous blue light (cB, 100 μmol m^{-2} s^{-1}) or continuous red light (cR, 100 μmol m^{-2} s^{-1}), for 5 days. Subsequently, they were subjected to a heat stress treatment at 44°C for 0, 4, or 5.5 hours at day-5, followed by a recovery period of 5 days at 22°C under respective growth light conditions.

(B) Representative thermotolerance phenotypes of the indicated genotypes are displayed.

(C) Quantification of the survival rates of seedlings in (B). The data are presented as the mean ± SD of at least three biological replicates, with approximately 50 plants per genotype examined in each biological replicate. Different letters indicate statistically significant differences between genotypes within each treatment (one-way ANOVA followed by Tukey's multiple comparisons test, $p < 0.05$).

Supplemental Figure 10

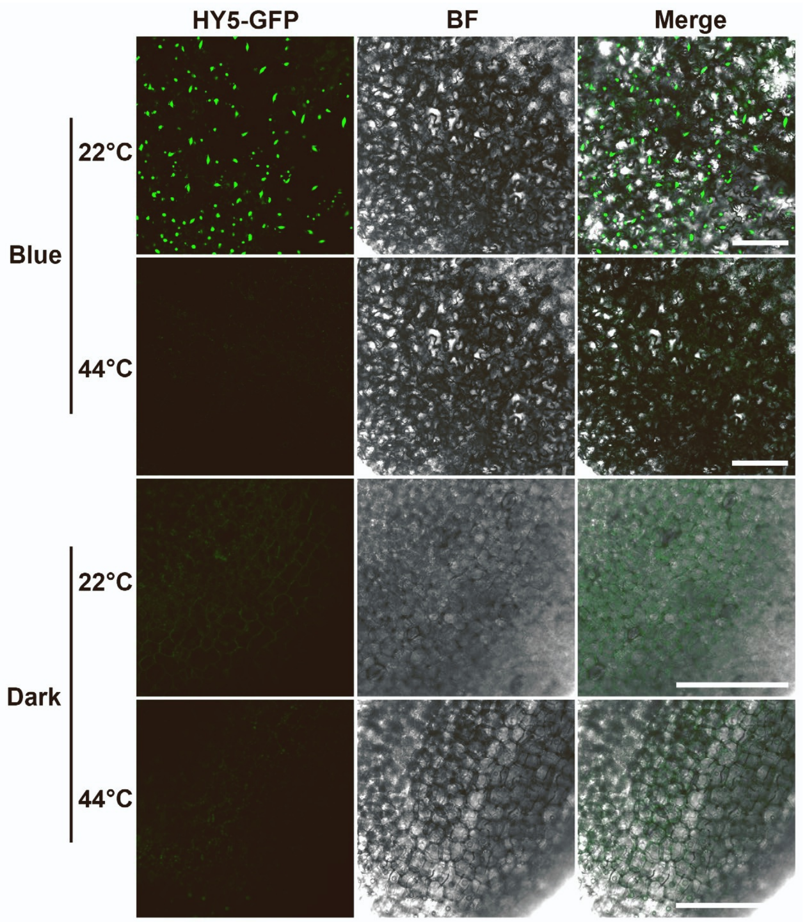

Supplemental Figure 10. Confocal images displaying the reduced HY5 GFP fluorescence in response to heat stress in cotyledon.

4-day-old *proHY5::HY5-GFP* seedlings were grown either in continuous darkness or blue light (100 μmolm^{-2}s^{-1}) at 22°C, and then subjected to heat stress treatment at 44°C for 4 hours, followed by fixing in 4% paraformaldehyde before imaging. Scale bar, 100 μm.

Supplemental Figure 11

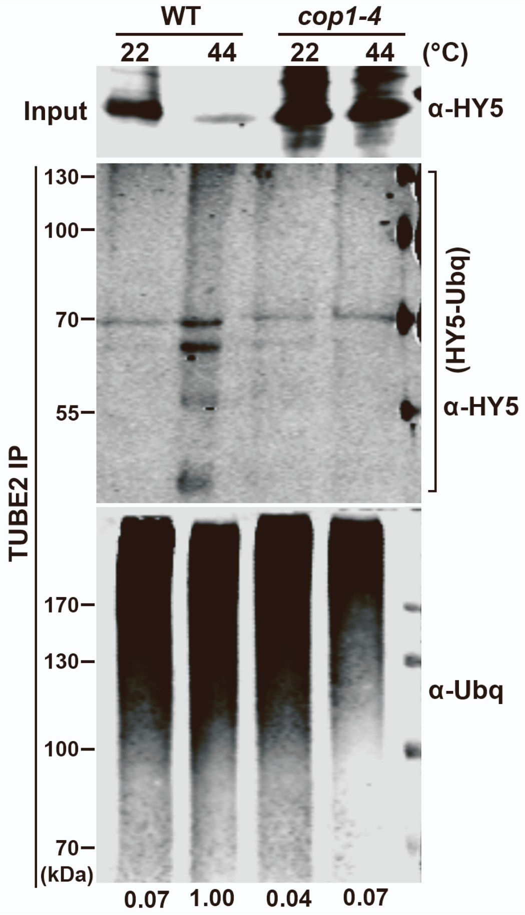

Supplemental Figure 11. COP1 is responsible for HY5 ubiquitination under heat stress.

Immunoblots showing the ubiquitination of endogenous HY5 in WT and cop1 mutants. 10-day-old WT and *cop1-4* seedlings grown at 22°C under blue light (100 μmol m^{-2} s^{-1}) were treated at 22°C or 44°C for 4 hours before sample collection. Total ubiquitinated proteins were purified by TUBE2-conjugated beads. Immunoprecipitated proteins were analyzed by immunoblots probed with anti-ubiquitin antibody (α-Ubq) or anti-HY5 antibody (α-HY5). HY5-Ubq indicates polyubiquitinated HY5. Level of ubiquitination was calculated as $[\text{HY5-Ubq intensity}]^{IP}/[\text{Ubq intensity}]^{IP}$. The level of HY5 ubiquitination at 44°C in WT were set to 1. The extent of HY5 ubiquitination relative to level of HY5 ubiquitination in WT at 44°C was shown below the immunoblots.

Supplemental Figure 12

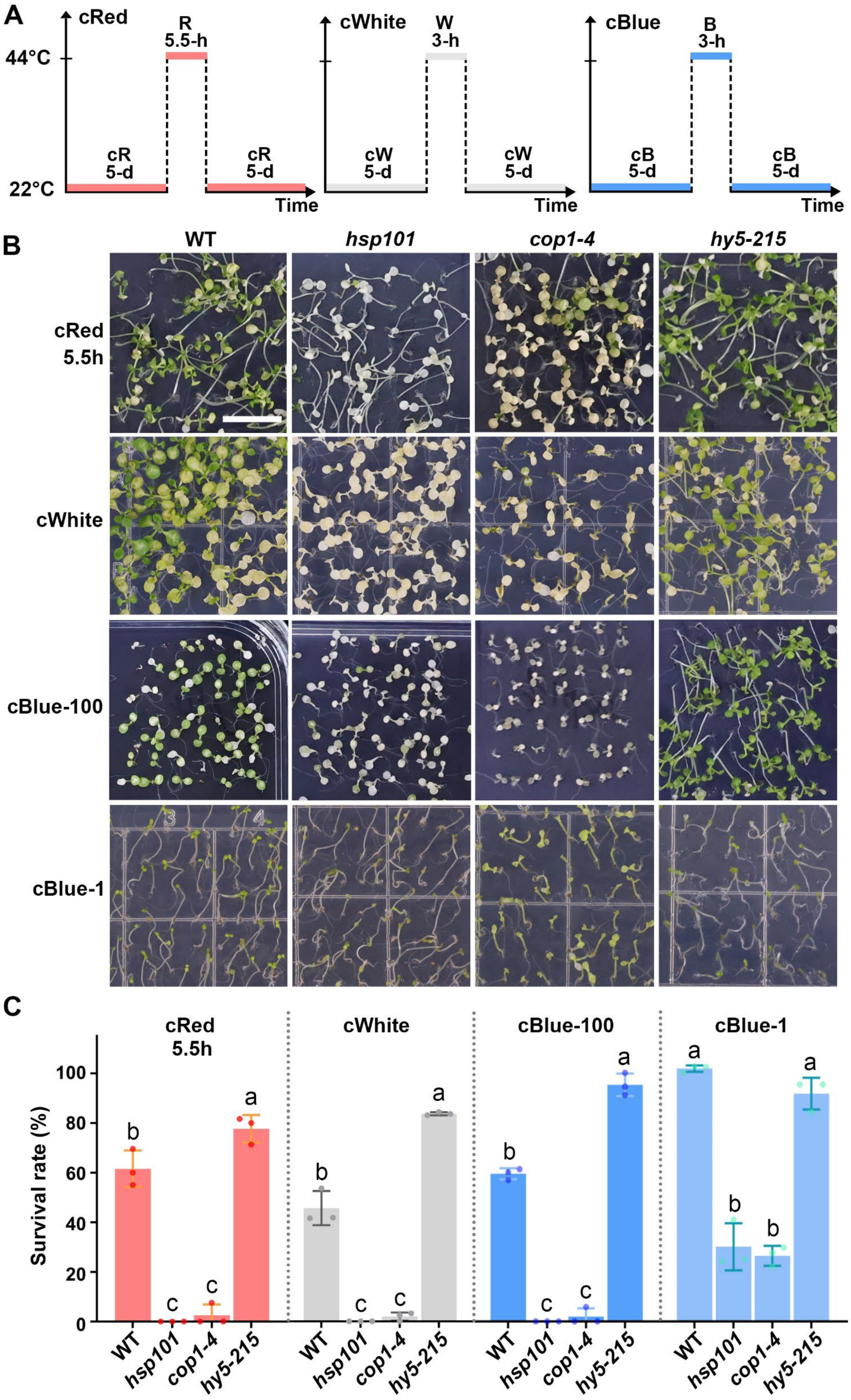

Supplemental Figure 12. The thermotolerance phenotypes of *cop1* and *hy5* mutants under continuous light conditions.

(A) Representative charts showing the heat stress experiments under continuous light conditions. Plants were initially grown on MS plates at 22°C under different light conditions, continuous blue light (1 μmol m^{-2} s^{-1} and 100 μmol m^{-2} s^{-1}), continuous white light (100 μmol m^{-2} s^{-1}) or continuous red light (100 μmol m^{-2} s^{-1}), for 5 days. Subsequently, they were subjected to a heat stress treatment at 44°C at day-5, followed by a recovery period of 5 days at 22°C under respective growth light conditions.

(B) Representative thermotolerance phenotypes of the indicated genotypes under continuous light conditions. Photos were taken after 5 days of recovery. cBlue-100, continuous 100 μmol m^{-2} s^{-1} of blue light; cBlue-1, continuous 1 μmol m^{-2} s^{-1} of blue light, cWhite, continuous white light (100 μmol m^{-2} s^{-1}); cRed, continuous red light (100 μmol m^{-2} s^{-1}). Scale bar, 1 cm.

(C) Quantification of the survival rates of seedlings in (B). The data are presented as the mean ± SD of three biological replicates, with approximately 50 plants per genotype examined in each biological replicate. Different letters indicate statistically significant differences between genotypes within each treatment (one-way ANOVA followed by Tukey's multiple comparisons test, $p < 0.05$).

The thermotolerance phenotypes and quantification date of cBlue-1, cBlue-100, cWhite for WT and *hsp101* mutants in (B) and (C) were taken from Figure 1, as the different genotypes in Figure 1 and this figure were performed and assayed simultaneously.

Supplemental Figure 13

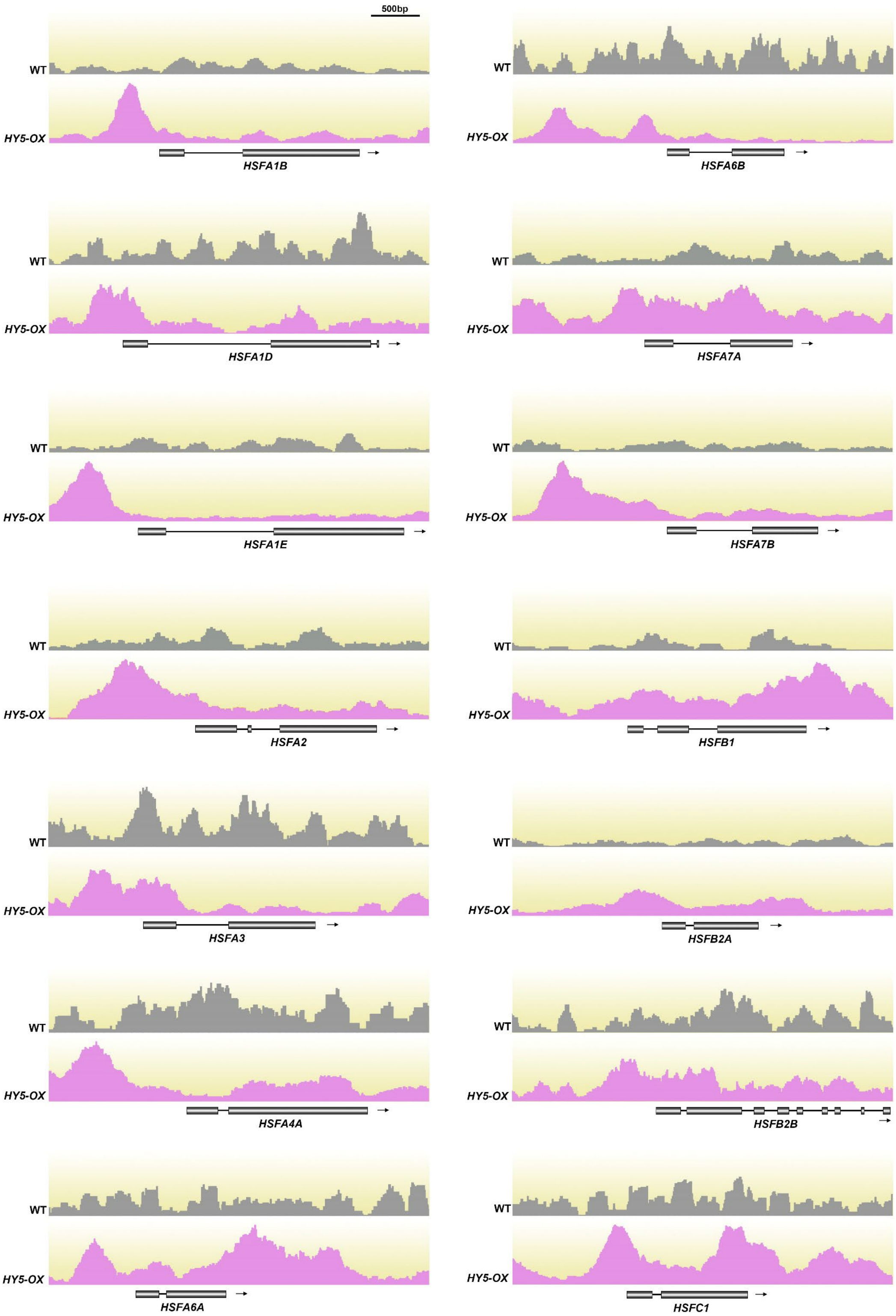

Supplemental Figure 13. Snapshots displaying the binding of HY5 to the promoters of *HSF* genes.

ChIP-seq data were obtained from Burko et al., 2020.

Supplemental Figure 14

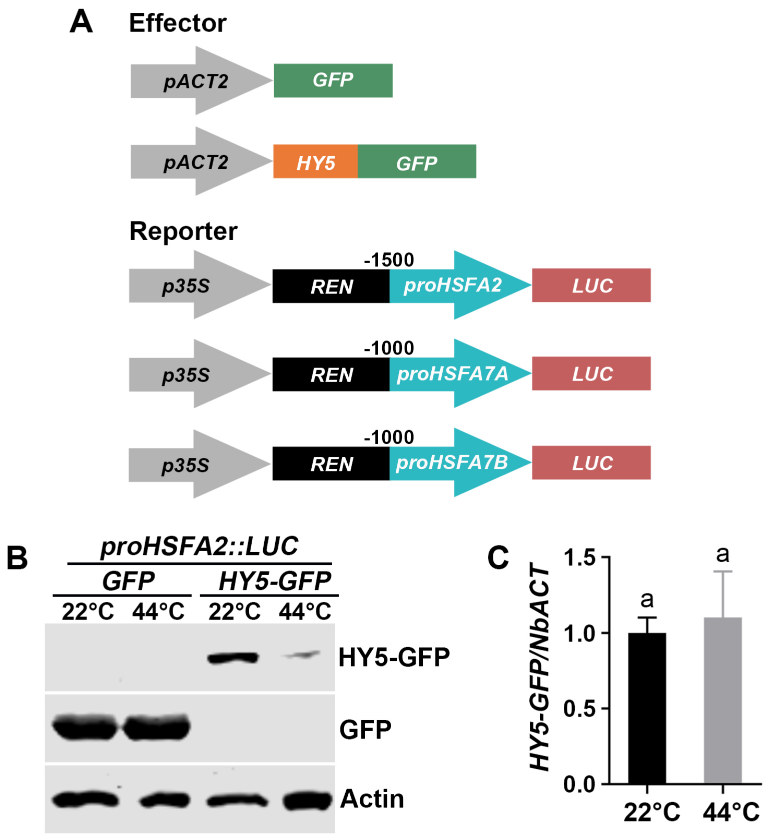

Supplemental Figure 14. The expression of HY5-GFP in tobacco transient experiments in Figure 7.

(A) Schematic representation of the effector and reporter plasmids employed in dual-LUC assays. The numbers (-1500 and -1000) indicate the positions of the *HSF*s promoters.

(B) Immunoblots showing the expression levels of HY5-GFP protein in tobacco transient expression assays conducted in Figure 7a. Tissues were collected from leaves subjected to co-transformation with *proHSFA2::LUC* and either *HY5-GFP* or *GFP*. The levels of HY5-GFP and GFP were detected using anti-GFP antibody. The levels of Actin were detected using anti-Actin 2 antibodies from Arabidopsis.

(C) RT-qPCR results showing the expression levels of *HY5-GFP* mRNA in (b). The mRNA levels of *HY5-GFP* were assessed using primers designed to target both the *HY5* and *GFP* regions. The qPCR signals are normalized by that of the Actin in tobacco (*NbActin*). The relative expression of the *HY5-GFP* at 22°C are set to 1. Significant differences was determined by a One-Way ANOVA test.

Supplemental Figure 15

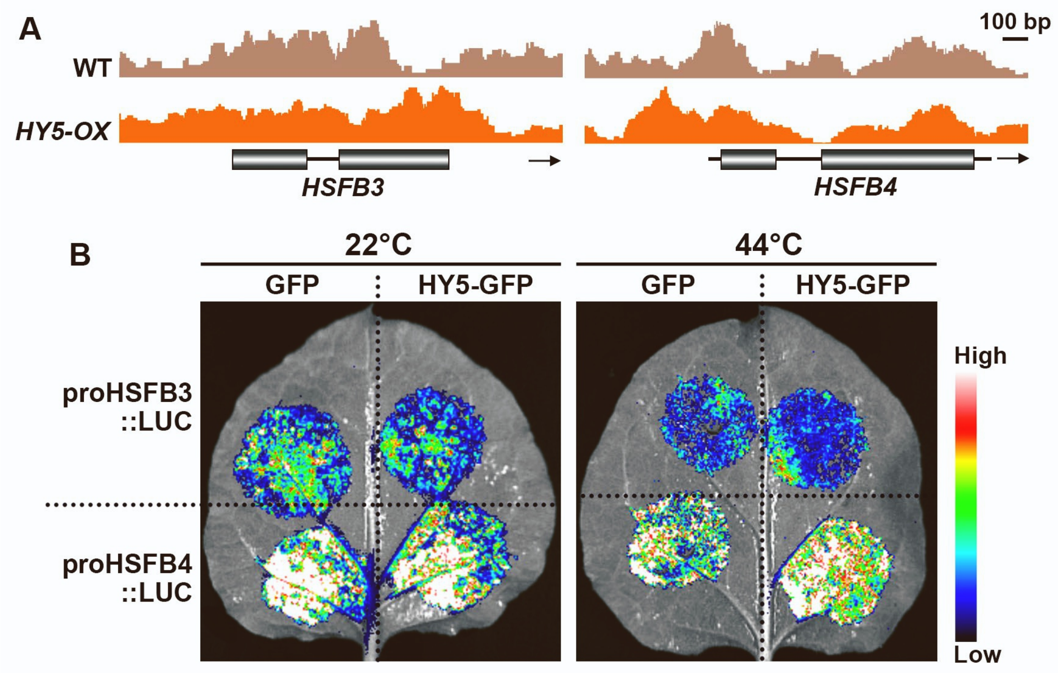

Supplemental Figure 15. HY5 does not regulate the expression of *HSFB3* and *HSFB4*.

(A) Snapshots showing that no significant binding of HY5 to the promoters of *HSFB3* and *HSFB4.*

(B) Dual-LUC assays showing the regulation of HY5 on *HSFB3* and *HSFB4* transcription. The Agrobacterium harboring effector and reporter plasmids were co-injected into tobacco leaves. Luciferase activities were evaluated using a CCD camera 72 hours after transfection. The plants were subjected to treatments at 22°C or 44°C for 1 hour before imaging.
